# Metal–hydrogel chelation interfaces for ultrasoft and bidirectional bioelectronics

**DOI:** 10.1093/nsr/nwaf399

**Published:** 2025-09-18

**Authors:** Yuyao Lu, Ziguan Jin, Yihui Jian, Depeng Kong, Hao Zhou, Yuhong Xu, Ruijue Cao, Zhuoheng Xia, Fan Yang, Qianglong Wu, Yang Gao, Aoran Cui, Shikuan Yang, Nenggan Zheng, Junhyuk Bang, Geng Yang, Seung Hwan Ko, Huayong Yang, Kaichen Xu

**Affiliations:** State Key Laboratory of Fluid Power and Mechatronic Systems, School of Mechanical Engineering, Zhejiang University, Hangzhou 310027, China; State Key Laboratory of Fluid Power and Mechatronic Systems, School of Mechanical Engineering, Zhejiang University, Hangzhou 310027, China; State Key Laboratory of Fluid Power and Mechatronic Systems, School of Mechanical Engineering, Zhejiang University, Hangzhou 310027, China; State Key Laboratory of Fluid Power and Mechatronic Systems, School of Mechanical Engineering, Zhejiang University, Hangzhou 310027, China; State Key Laboratory of Fluid Power and Mechatronic Systems, School of Mechanical Engineering, Zhejiang University, Hangzhou 310027, China; State Key Laboratory of Fluid Power and Mechatronic Systems, School of Mechanical Engineering, Zhejiang University, Hangzhou 310027, China; Center for Plastic & Reconstructive Surgery, Department of Stomatology, Zhejiang Provincial People’s Hospital, Affiliated People’s Hospital, Hangzhou Medical College, Hangzhou 310014, China; School of Stomatology, Zhejiang Chinese Medical University, Hangzhou 310053, China; Center for Plastic & Reconstructive Surgery, Department of Stomatology, Zhejiang Provincial People’s Hospital, Affiliated People’s Hospital, Hangzhou Medical College, Hangzhou 310014, China; Center for X-mechanics, Department of Engineering Mechanics, Zhejiang University, Hangzhou 310027, China; Center for X-mechanics, Department of Engineering Mechanics, Zhejiang University, Hangzhou 310027, China; Institute for Composites Science Innovation, School of Materials Science and Engineering, Zhejiang University, Hangzhou 310027, China; Institute for Composites Science Innovation, School of Materials Science and Engineering, Zhejiang University, Hangzhou 310027, China; State Key Laboratory of Brain-Machine Intelligence and the Qiushi Academy for Advanced Studies, Zhejiang University, Hangzhou 310027, China; Department of Mechanical Engineering, Seoul National University, Seoul 08826, Republic of Korea; State Key Laboratory of Fluid Power and Mechatronic Systems, School of Mechanical Engineering, Zhejiang University, Hangzhou 310027, China; Zhejiang Key Laboratory of Intelligent Robot for Operation and Maintenance, Hangzhou 310000, China; Department of Mechanical Engineering, Seoul National University, Seoul 08826, Republic of Korea; State Key Laboratory of Fluid Power and Mechatronic Systems, School of Mechanical Engineering, Zhejiang University, Hangzhou 310027, China; State Key Laboratory of Fluid Power and Mechatronic Systems, School of Mechanical Engineering, Zhejiang University, Hangzhou 310027, China

**Keywords:** hydrogel, metal, chelation, hybrid interfacial bonding, bioelectronics

## Abstract

Emerging demand in soft bioelectronic systems poses critical challenges in stiffness control and end-to-end connections due to the huge modulus difference in various components. Here, a bidirectional electrical interface of hydrogel and metal electrodes to bridge soft skin/tissue and data collection circuits is enabled by coordination interactions. The dual-mode chelation including internal chelation and surface chelation effectively configures the cross-linking structure of hydrogel, as well as enhances the binding interface of metal–hydrogel complex surfaces. Internally, strong chelation competes with esterification, yielding tissue-like softness of hydrogel with an ultra-low modulus of ∼339.9 Pa. Externally, the hydrogel passivates the combined metal surfaces, promoting the formation of interlocked structures between metal oxide nanoislands, achieving a high binding strength of ∼1.95 MPa without compromising electrical conductivity. The stable electrical interconnections via hybrid interfacial bonding enable high signal-to-noise ratio signal recordings from the skin, neural surfaces and brain, maintaining reliable performance, even under mechanical disturbances. This work provides an effective strategy for achieving mechanically and electrically robust hybrid bioelectronic interfaces, advancing their applications in capturing both *in vitro* and *in vivo* electrical signals.

## Teaser

This study develops a hybrid bioelectronic interface using hydrogel and metal electrodes, ensuring both mechanical flexibility and electrical stability for reliable in vivo and in vitro signal recording. (28 words)

## INTRODUCTION

Soft electrodes, especially hydrogels, are highly desired for high-fidelity electrophysiological signal recording due to tunable mechanical and charge transport properties [1,2]. Similar to biological organs and tissues, the Young’s modulus of hydrogel ranges from tens to tens of thousands of pascals, depending on cross-linking density, concentration of polymers etc. [3]. Compared to on-skin measurements, the critical challenge for *in vivo* detection is to design a hydrogel with tissue-like softness, mechanical robustness and high conductivity, as well as superior interfacial adhesion [4–6]. However, most hydrogels with simple networks generally suffer from fragility and relatively low electrical conductivity [7,8].

A representative example is poly(3,4-ethylenedioxythiophene):poly(styrene sulfonate) (PEDOT:PSS), which exhibits relatively high conductivity (>20 S/m) but possesses a modulus over 10^3^ times higher than that of soft tissues [9]. Although the softness and adhesion of hydrogels are able to be modulated via building multiple network interactions, especially the physical cross-linking networks, hydrogels still display mechanical mismatches with flexible or rigid acquisition systems. This results in fast device failure under mechanical interference due to weak interfacial interactions [8]. Commercially, such issues have been addressed by immobilizing the hydrogel surroundings or configuring interlocking button structures, restricting shear displacement at the skin–hydrogel interface [10].

To realize soft–rigid hybrid electronics, significant efforts have been dedicated to constructing stretchable transistors and circuits using stiffness modulation, buckling strategies, structural engineering, interfacial combination engineering and intrinsic stretchability engineering [11–14]. These technologies enable reasonable conductive stretchability in soft–rigid hybrid electronics. Despite these innovative strategies, achieving stable connections between soft hydrogels and flexible metal electrodes remains a challenge due to the presence of solvents on the hydrogel surface, which weakens adhesion and electrical contact [15].

Herein, we propose a soft hydrogel electrode with tissue-like modulus (∼339.9 Pa) and high conductivity (19.4 S/m), which forms stable electrical connections with flexible/elastic metal electrodes through elastic (E)–elastic (E) and elastic (E)–flexible (F) binding. The modulus of hydrogel was effectively regulated by internal chelation of phytic acid (PA) with metal ions, during which coordination interactions serve as sacrificial bonds, competing with the esterification reaction to dissipate energy within the double network (DN) hydrogel. Meanwhile, this internally chelated hydrogel can also be mechanically and electrically connected to metal foils or films through surface chelation. Strong surface binding strength, driven by the interlocking nanoisland structure (∼0.6 μm height) formed between two Cu foils (100 μm), achieves a strength of 1.95 MPa. Such a stable electrical binding interface enabled by surface chelation is also suitable for many other metals including physically deposited Au films and liquid metals (LMs).

When the soft hydrogel, attached to skin or tissue, forms a bridge with elastic or flexible metal interfaces, the resulting hybrid bioelectronic system, designed with a gradient modulus, enables high signal-to-noise ratio (SNR) recordings of electrophysiological signals, including electromyographic, neural and electrocortical signals. As a result, continuous and clear action potentials were recorded from muscle tissue and the sciatic nerve using this hybrid system under both rest and stimulation conditions.

## RESULTS AND DISCUSSION

### Design of soft and bidirectional bioelectronic interfaces

To obtain high-fidelity electrophysiological signal acquisition, a DN hydrogel composed of polyvinyl alcohol (PVA), PA and honey (PPH) was constructed using the method of our previous study [16]. The three components are considered as safe and biocompatible materials recognized by the U.S. Food and Drug Administration [17–19]. PVA, a highly hydrophilic polymer, participates in both the formation of chemical networks and physical networks through forming ester bonds and hydrogen bonds with PA and honey [20]. PA not only serves as the chemical cross-linker to form a supramolecular structure with PVA, but also contributes to the ionic conductivity of the hydrogel by providing mobile protons and phosphate groups [21]. Honey, which is rich in saccharides and amino acids, primarily facilitates the formation of physical networks by establishing strong hydrogen bonds with both PVA and PA, thereby enhancing the flexibility and viscosity of hydrogels [22].

**Figure 1.**
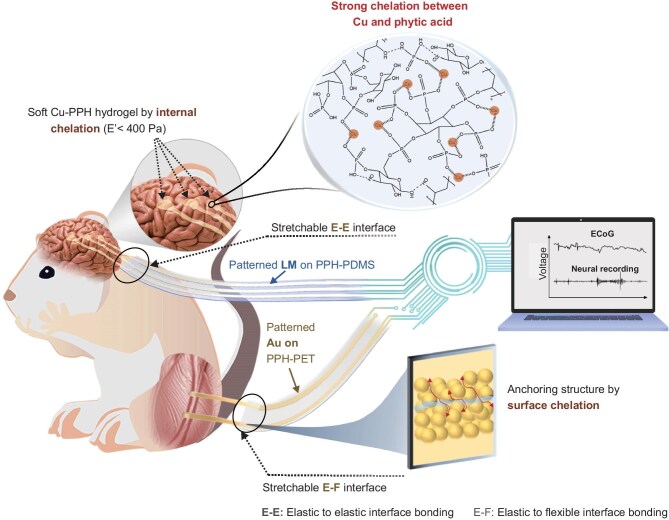
Concept of internally chelated soft hydrogels, bidirectional E–E and E–F interfaces enabled by surface chelation of PPH hydrogel with metals, designed for high-fidelity electrophysiological signal recording. The bidirectional electrical interface refers to the stretchable interface bridged by the internally chelated soft hydrogel and the externally chelated metal–hydrogel electrode. The former is connected to a soft tissue, while the latter is connected to a flexible or rigid circuit board, ensuring a stable electrical connection.

Based on the aforementioned structures, PPH hydrogel is endowed with good mechanical strength, ionic conductivity and bio-adhesive properties. To optimize the stability of electrical contact between hydrogel and skin/tissue, the chelation reaction of metal ions and PA was incorporated into this hydrogel, which effectively reduced the Young’s modulus of hydrogel and further enhanced the ionic conductivity. Specifically, the coordination interactions between PA and metal ions act as sacrificial bonds to dissipate the energy of PPH hydrogel through competing with the esterification reaction. This allows lower stiffness but higher mechanical stability of PPH hydrogel. Thus, a soft hydrogel with a modulus of about 339.9 Pa can be achieved by chelating PA with ionized Cu nanoparticles (Cu^2+^), and is applied as soft and stable contact electrodes for interfacing with brain tissues (Fig. 1).

On the other hand, PPH hydrogels containing PA also present highly significant chelation behaviors with the surface of metals. By depositing Au nanoparticles or printing the LM nanoparticles onto the PPH film, strong interfacial adhesion can be formed under surface chelation. Significantly, the surface-chelated Au–PPH on a flexible polyethylene terephthalate (PET) film and LM–PPH on an elastic polydimethylsiloxane (PDMS) film can be mechanically and electrically connected with the soft Cu–PPH hydrogel to form stretchable E–F and E–E interfaces, respectively. By constructing mechanically matched and highly conductive binding interfaces that bidirectionally connect to both skin/tissues and data collection systems, electrophysiological signals with clear baselines and high SNR can be stably tracked.

### Modulus-regulable biomimetic hydrogels induced by internal chelation

**Figure 2.**
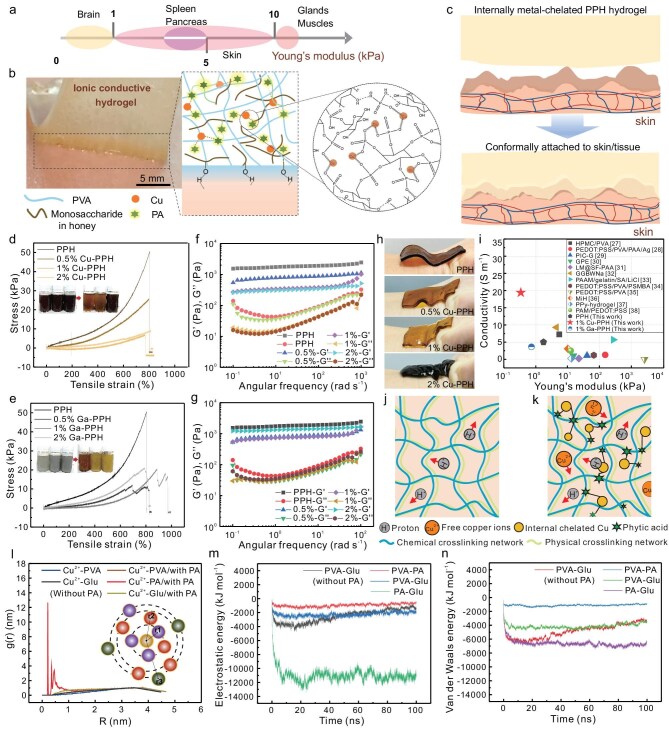
Internal chelation-enabled soft hydrogels with tissue-like modulus. (a) Different moduli of natural soft tissues and organs. (b) Photo and structural illustration of a Cu-chelated PPH hydrogel attached onto skin. (c) Schematic of a metal-chelated hydrogel with tissue-like softness that can be conformally attached to skin. (d and e) Tensile performances of Cu-chelated PPH hydrogel and Ga-chelated PPH hydrogel with different contents of Cu nanoparticles and Ga_2_O_3__–_LM microspheres, respectively. The inset photos illustrate the changes in hydrogel solution with varying metal ion concentrations before and after internal chelation. (f and g) Changes in storage modulus (G′) and loss modulus (G′′) of Cu–PPH and Ga–PPH hydrogels with different contents of Cu nanoparticles and LM microspheres as a function of angular frequency. (h) Photos of Cu–PPH hydrogels with different moduli mechanically adapted onto the deformed wrist skin. (i) Comparison of the modulus and conductivity of pristine PPH, 1% Cu–PPH and 1% Ga–PPH hydrogels with those reported in previous studies. (j and k) The ionic conductive mechanisms of pristine PPH and Cu–PPH hydrogels. (l) RMSD results of glucose in the presence and absence of PA within 100 ns. (m and n) Electrostatic energy and van der Waals energy calculated for both systems without/with PA in 100 ns.

For reliable electrophysiological signal recording, tuning the Young’s modulus of bioelectrodes is critical to ensure conformal, stable contact with soft, dynamic tissues, complementing conductivity and adhesion. Mechanical matching minimizes interfacial gaps and motion artifacts, thereby enhancing signal fidelity and improving the SNR. As one of the candidates for bioelectronic interfaces, PPH hydrogel with DN can be tailored to mimic the modulus of natural tissues and organs by internal chelation between PA and metal ions. Via regulating the cross-linking networks, hydrogels are able to be fabricated in suitable softness, and their moduli are close to those of skin, spleen, pancreas and muscle, ranging from 1 to 10 kPa (Fig. 2a). Brain tissue is the softest and most fragile part of the human body, and the modulus is only distributed in hundreds of pascals. It is challenging for either natural polymers or synthetic polymers to obtain both brain-like softness and toughness without structural optimizations. Compared to single network (SN) hydrogels, DN hydrogels are endowed with ultrahigh mechanical strength and toughness due to the high density of interpenetrating networks. By incorporating coordination interactions between PA and metal ions (i.e. Cu^2+^) as sacrificial bonds within the PPH hydrogel structure, the rigid chemical networks can be partially replaced by polydentate complexes, enhancing flexibility and toughness (Fig. 2b) [23]. Therefore, the internally chelated Cu–PPH hydrogel with high softness and adhesion can be conformally attached to the surface of skin/tissue (Fig. 2c, Fig. S1).

To verify chelation-induced mechanical optimizations for PPH hydrogels, Cu nanoparticles (diameter: 30–50 nm) and Ga_2_O_3_ microspheres (diameter: 100–650 nm) were dispersed in deionized (DI) water and used as chelating metal ions at various valence states (Fig. S2). To figure out the optimal parameters for modulus regulation of PPH hydrogel, 0.5%, 1% and 2% Cu nanoparticles and Ga_2_O_3_ microspheres were selected for investigations. As shown in Fig. 2d and e, significant decreases in fracture stress of about 25 and 38 kPa were observed for Cu–PPH and Ga–PPH hydrogels, respectively, as the content of Cu^2+^ and Ga^3+^ increased from 0% to 0.5%. As the content increased to 1%, the stress of Cu–PPH at the maximum strain further decreased from about 25 to 7 kPa, and eventually obtained a saturated value of about 5 kPa when the content was 2% (Fig. 2d). The Ga–PPH hydrogels exhibited significant energy dissipation within the cross-linking structure as the content of ionized Ga_2_O_3_ microspheres (Ga^3+^) increased from 0.5% to 1%. This can be attributed to the coordination interactions between PA and Ga³⁺, which led to an increase in fracture strain from 800% to 1000% (Fig. 2e). Meanwhile, the stress of the Ga–PPH hydrogel decreased at strains below 800%, indicating a reduction in Young’s modulus. However, at around 2% Ga₂O₃, a partial saturation of chelation sites occurs, and the excess Ga³⁺ ions may act as physical cross-linking nodes or induce localized ionic aggregation, which transiently enhances mechanical strength.

To intuitively show the modulus variations of PPH after chelation with different contents of Cu nanoparticles and Ga_2_O_3_ microspheres, rheological properties at different angular frequencies were characterized. The storage moduli of both Cu–PPH hydrogels and Ga–PPH hydrogels were higher than the loss moduli, implying that elastic deformation dominated in the stretching process. Remarkably, the storage moduli of different contents of Cu nanoparticles and Ga_2_O_3_ microspheres in PPH hydrogels displayed consistent change with the stress variations under tensile strain (Fig. 2f and g). Obviously, 1% Cu–PPH and 1% Ga–PPH were observed to have the lowest elasticity moduli, which are 339.9 and 730.6 Pa at an angular frequency of 1 rad/s, respectively. The increasing full width at half-maximum peaks in the infrared (IR) spectra of 1% Cu–PPH and 1% Ga–PPH indicated the enhanced intramolecular interactions due to the internal chelation (Fig. S3) [24]. Thus, the obtained 1% Cu–PPH and 2% Cu–PPH hydrogels can be conformally attached onto deformed wrist skin (Fig. 2h). Furthermore, due to the internal chelation, 1% Cu–PPH hydrogel also presented the fastest healing speed after laser cutting (Fig. S4) [25].

In addition, the conductivity of pristine PPH, 1% Cu–PPH and 1% Ga–PPH hydrogels was investigated. The 1% Cu–PPH hydrogel exhibited nearly four times higher conductivity (19.4 S/m) compared to pristine PPH (5 S/m) and 1% Ga–PPH (3.6 S/m). Compared with previously reported hydrogels, the 1% Cu–PPH hydrogel developed in this work exhibits a lower Young’s modulus and higher conductivity, which are favorable for forming a more robust conductive biointerface (Fig. 2i) [26–37]. According to the Grotthuss mechanism, the ionic conductivity of pristine PPH hydrogel primarily depends on the migration of free protons within the hydrogel (Fig. 2j) [38]. When PA chelates with Cu²⁺, a dynamic ionic network containing both protons and Cu²⁺ is formed, resulting in a significant enhancement in the ionic conductivity of Cu–PPH hydrogels (Fig. 2k) [39,40]. In contrast, Ga³⁺ exhibits a stronger hydration effect with a higher charge density, leading to a larger and more tightly bound hydration shell that significantly hinders its mobility [41,42]. Thus, the 1% Cu–PPH hydrogels present higher conductivity than 1% Ga–PPH hydrogels.

Besides, carbon nanotube (CNT), as a common dopant, was also chosen for comparison in mechanical optimization with that of metal ions in PPH hydrogels. Different from the chelation reaction, the doping of CNTs in the PPH hydrogel was observed, with evident improvement in mechanical strength (Fig. S5). To investigate the universality of modulus regulation by using this method, 1% Ag nanowires, 1% Cu nanoparticles and 1% Ga_2_O_3_ microspheres were chosen as coordinators for internal chelation with PA in PPH hydrogels. The evident reduction in fracture strain of Ag–PPH, Cu–PPH and Ga–PPH confirmed the strong chelating ability of PA with different metal ions. Owing to the relatively low modulus and high conductivity, metal-chelated PPH hydrogels showed lower contact impedances on skin than either dry Cu electrode or commercial gel at frequencies ranging from 100 Hz to 100 kHz (Fig. S6).

As reported in our previous work, PPH with a high content of PA (42 wt%) was endowed with low cytotoxicity and an antibacterial property due to the unique components and highly cross-linked networks. Via chelating with Cu^2+^ and Ga^3+^, the 1% Cu–PPH and 1% Ga–PPH with 22 wt% of PA both presented clear inhibition zones after 24 h of culture at 37°C. Obviously, the Ga–PPH hydrogel presented better bacteriostasis than that of PPH and Cu–PPH in 2 weeks, due to the higher concentration of positive ions inside the polymer network (Fig. S7).

Molecular dynamics (MD) simulations were conducted to reveal the non-bonding interactions for two hydrogel systems. The hydrogel system without PA was observed with slight changes in spatial structure after 60 ns of simulation except the spontaneous aggregation of glucose and PVA chains under electrostatic interactions (Fig. S8a–d). However, real-time conformational screenshots of the hydrogel system with PA reflect high intermolecular interactions and fast aggregations of PVA, glucose, PA and Cu^2+^ in only 20 ns. After that, the structure of this system reached an equilibrium state (Fig. S8e–h). The enlarged screenshots and radial distribution function (RDF) results revealed that the Cu^2+^ nanoparticles were surrounded by a large number of PA molecules (Fig. 2l, Fig. S8i). In addition to PA, glucose was a molecule that was highly distributed around Cu^2+^. In both systems, RDF values indicated that PVA molecules were mainly surrounded by glucose and PA due to their rich hydroxyl groups. It can be seen that root-mean-square deviation (RMSD) of the hydrogel system with PA was lower than that without PA, indicating fewer fluctuations and higher stability of the system (Fig. S8j and k).

Obviously, PA was surrounded by Cu^2+^, glucose and PVA in order of distance (Fig. S9). After adding PA molecules, the system was easier to be stabilized, so the kinetic energy of the system was increased and potential energy was decreased (Fig. S10a and b). Owing to rich hydroxyl groups, the electrostatic interaction and van der Waals interaction energy between glucose and PA were observed, with the highest value of about −11 000 and −6600 kJ/mol, respectively (Fig. 2m and n, Fig. S10c and d). Due to the esterification reaction between PVA and PA, the non-bonded interaction between them is relatively low. Overall, the simulation results not only highlight the crucial role of PA in establishing the DN structure of the hydrogel, but also provide valuable insights in coordination interactions between Cu²⁺ and PA molecules within the hydrogel system.

### Heterogeneous osmotic interface chelation-induced interlocking structures

**Figure 3.**
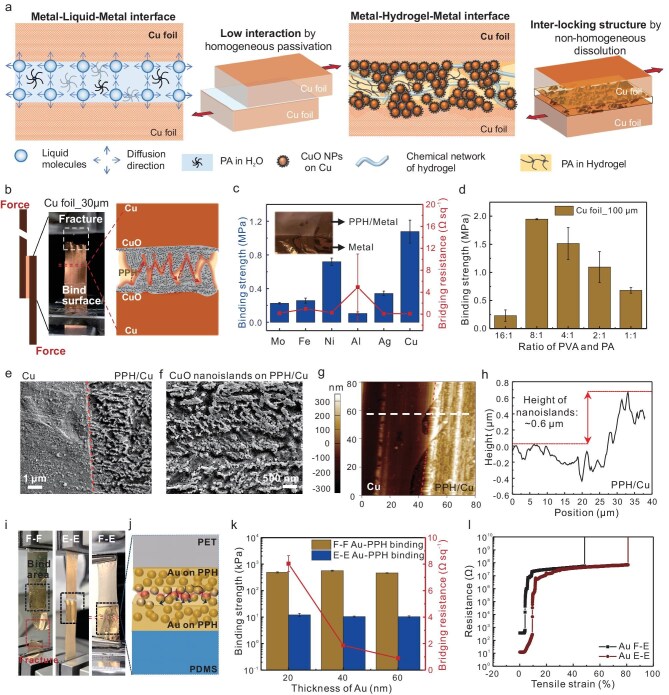
Surface chelation-enhanced binding interface between metals. (a) Interfacial binding strength of liquid (PA)-passivated metal surfaces and semi-solid (PPH)-passivated metal surfaces induced by surface chelation. (b) High lap shear strength of interlocked surface of two Cu foils with thickness of 30 μm achieved by surface chelation. (c) Adhesion strength and bridging resistance of different metal foils under surface chelation. Data are presented as mean ± SD (*n* = 3). (d) Changes in adhesion strength of Cu foils chelated by PPH hydrogels with varied ratios of PVA and PA. Data are presented as mean ± SD (*n* = 3). (e) SEM images of pristine Cu (left side of dotted line) and PPH-treated Cu (right side of dotted line). (f) SEM image of CuO nanoislands on PPH-treated Cu surface. (g) AFM image of pristine Cu (left side of dotted line) and PPH-treated Cu (right side of dotted line) (h) Height difference between the pristine Cu and PPH-treated Cu, measured along the white dashed line. (i and j) Photo images of F–F, E–E and E–F binding interfaces of Au nanoparticles on stretchable PPH–PDMS (E) and flexible PPH–PET (F). (k) Statistical results of binding strength and bridging resistance of F–F and E–E interfaces with different thicknesses of Au, i.e. 20, 40 and 60 nm. Data are presented as mean ± SD (*n* = 3). (l) Resistance changes of F–E, E–E binding interfaces of Au electrodes as a function of tensile strain.

Based on MD simulations, the PPH hydrogel exhibits strong potential as an interfacial chelating layer for a wide range of metal foils. This behavior is consistent with the well-established industrial use of PA as a corrosion inhibitor, where it functions as an effective surface passivator for metals [43]. In the molecular structure of PA, four phosphate groups are in the same plane. Therefore, it is easy to form a dense single-molecule protective film on the metal surface, which can effectively prevent O_2_ or chemicals from the ambient environment [44]. Such molecular-scale surface coverage is critical for long-term metal protection and is harnessed in our system to stabilize metal–hydrogel interfaces. When PA is applied as a solution in a sandwich structure, it tends to induce homogeneous passivation on both metal surfaces. This uniform surface modification limits the formation of interfacial features, leading to weaker mechanical interlocking and reduced surface interactions. However, as PA is contained in a hydrogel system, it plays two main roles in forming DNs with PVA via the esterification reaction, as well as hydrogen bonding with glucose in honey and water. Significantly, the semi-solid state of hydrogel endows it with enough space and time for coarsening of metal surfaces by the freestanding PA molecules outside the hydrogel networks [45,46]. Such heterogeneous surface dissolution and passivation lead to the strong interlocking structure due to the *in situ* formation of oxidized nanoislands on two metal surfaces (Fig. 3a) [47,48]. Also, PPH hydrogels with abundant hydroxyl groups are able to adhere strongly to the roughened and enlarged surface of these nanoislands. Notably, the strong binding strength formed between two Cu billets (thickness: 1 cm) by surface chelation within 72 h can be detached by compressing stress of about 33 MPa (Fig. S11, Supplementary Video 3).

When a thin PPH film was coated between two Cu foils, the alternating distributed nanoislands generated on surfaces were strongly interlocked (Fig. 3b). The strong binding strengths between two pieces of Cu foils were measured by lap shear tests. It is worth noting that the Cu foil with a thickness of 30 μm was fractured, in contrast to the separation of the binding interface during the test when the thickness of PPH between the Cu foils was only 4.73 μm. Meanwhile, the bridging resistance between Cu foils was only 0.12 Ω/sq (Fig. S12). First, the passivation time was considered as one of the most critical issues to affect the binding strength. After 72 h of heterogeneous dissolution, the lap shear strength of two Cu foils with a thickness of 100 μm reached the highest value of 1.72 MPa. When the time was less than 48 h or over 120 h, the binding strength only achieved about 0.89 and 0.73 MPa, respectively (Fig. S13a). The insufficient passivation time for the formation of nanoislands resulted in the weak interlocking between Cu foils. However, too long a duration also affected the morphology of the nanoislands due to over-permeation of PA molecules, leading to reduction in binding strength. In contrast, the PA solution coated between two Cu foils with homogeneous interfacial dissolution was observed with no surface adhesion, even after 72 h. The strong adhesion strength formed between two Cu foils relied on both the passivation-induced interlocking structure of CuO nanoislands and the adhesion of PPH films.

According to the abovementioned principle, the interfacial chelation of PPH on other metal foils was also investigated by lap shear tests. The binding strengths for Mo, Fe, Ni, Al, Ag and Cu were 0.23, 0.26, 0.72, 0.1, 0.34 and 1.08 MPa, respectively (Fig. 3c). Similar to passivation reactions, the strong interfacial chelation of PPH on the (110) crystal planes of Ni and Cu foils facilitated the heterogeneous dissolution process, resulting in the high binding strengths. However, Al foils with a (110) crystal plane exhibited poor binding strength when coated with PPH due to their high chemical reactivity in an aqueous environment.

Although the resistance of the hydrogel was orders of magnitude higher than that of metals, the bridging resistance between the metal foils with a middle layer of PPH after transpassivation was close to the resistance of metal itself. Specifically, Mo, Fe, Ni, Ag and Cu all presented high binding strength with relatively low bridging resistance of below 1 Ω/sq even after 24 h of interfacial chelation with PPH. However, the Al foils were observed to show a remarkable resistance increase from 4.94 to 2762.25 Ω/sq after 24 h of reaction at room temperature (Fig. S13b). The concentration of PA in PPH was another critical parameter that influenced the adhesion strength. It can be seen that the binding strength of Cu foils was relatively high when the ratio of PVA and PA was 4:1 and 8:1 at about 1.51 and 1.95 MPa, respectively. As the PA content further increased, the cross-linking network of the hydrogel became denser, which indicates the low content of free PA molecules (Fig. S14). This result indicated that a suitable content of PA was required for transpassivation on Cu surface (Fig. 3d). The as-formed morphology and height of CuO nanoislands are mainly influenced by the chelation of free PA molecules inside the PPH network with the metal surfaces.

After selecting an optimal passivation time and PA content, the stable and strong binding interface between Cu foils was realized. The nanoislands generated on the Cu surface were clearly observed from the scanning electron microscope (SEM) images (Fig. 3e and f), which is highly distinguished from the surface morphology of pure Cu (left side of dotted line in Fig. 3e). These raised nanoislands were stacked with CuO nanoparticles, leading to the height of the nanoislands exceeding the plane of pure Cu for about 600 nm, as observed by atomic force microscopy (AFM) (Fig. 3g and h, Figs S15 and S16). Thus, the interlocked structure between two pieces of Cu foil with out-of-plane nanoislands can be achieved.

After the transpassivation, the existence of CuO nanoparticles on the nanoislands was identified by X-ray photoelectron spectroscopy (XPS) due to the appearance of strong satellite peaks of Cu^2+^, together with the strong peaks of vacancy oxygen (O_v_) and surface oxygen groups (–OH, O^2^^−^) (O_A_) (Fig. S17a and b). Significantly, the CuO nanoislands formed on the Cu surface can be further confirmed by the absence of Cu^2+^ and the appearance of strong O_v_ peaks in the XPS spectra of PPH-treated Cu after surface cleaning (Fig. S17c and d). Meanwhile, the higher intensity of the (111) plane than that of pristine Cu also provided the evidence of CuO after PPH treatment (Fig. S18).

As a kind of universal chelator for metals, PPH was used as the interlayer for deposition of Au nanomembrane by physical vapor deposition (PVD). The flexible (F) Au electrodes were obtained by depositing Au nanoparticles on PPH–PET. For elastic (E) Au electrodes, the PPH was spin-coated on the PDMS–PET substrates, and the PET substrate was peeled off during tests. By aligning the flexible Au electrodes with the elastic Au electrodes face to face, F–F, F–E and E–E binding interfaces, enabled by interlocking structures of two electrode surfaces with Au nanoparticles, were fabricated (Fig. 3i) [49]. Since the evaporation temperature (60°C–80°C) is close to the glass transition temperature (85.95°C), the PPH hydrogel enters a viscoelastic state during the PVD process, enabling partial penetration of Au nanoparticles into the hydrogel film (Fig. 3j). Notably, the F–F-connected interface of Au electrodes was observed with a strong lap shear strength of about 566.3 kPa (Fig. S19a, Supplementary Video 4). Meanwhile, the fracture strain of the three E–E binding interfaces with different thicknesses of Au films was over 90%, indicating the strong binding strength (Fig. S19b). This illustrated the strong chelation capability of PPH with Au nanoparticles. The bridging resistances of F–F interfaces of Au electrodes with thicknesses of 20, 40 and 60 nm were 8.0, 1.9 and 0.9 Ω/sq, respectively (Fig. 3k). The stretchable F–E and E–E interfaces of Au electrodes enable a conductive strain of about 4% and 10%, respectively (Fig. 3l).

### Friction passivation and anti-oxidation of LM on PPH surface

Highly stretchable and conductive LM-based electrodes have also been widely applied for soft bioelectronics. Due to the high surface tension, the large contact angle between LM and substrates is one of the critical obstacles for printing [50]. Existing printing methods have utilized the adjustable wettability of core-shell structured LM with superficial Ga_2_O_3_ films to realize high-resolution patterning [51]. However, the unresolved problem is that the printed LM electrodes are easily scratched by touching due to the fluidic property. For electrophysiological tests, the LM residues on skin/tissue may cause inflammation, as well as the failure of sensors.

**Figure 4.**
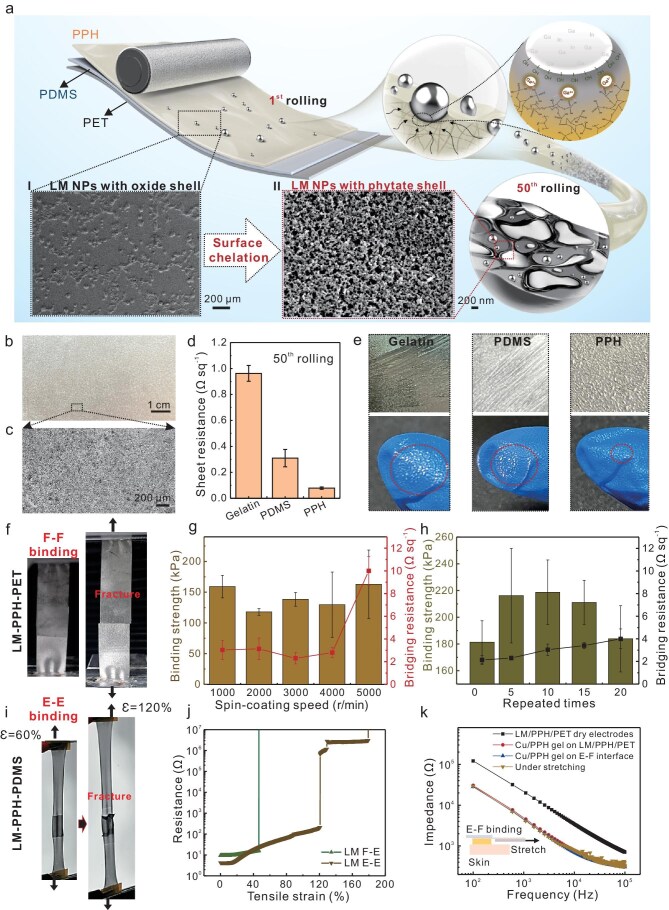
Surface chelation-enhanced binding interface of stretchable LM electrodes. (a) Schematic of the roll-printed LM on PPH hydrogel, where the dimensional structure of LM converts from LM microspheres to surface-passivated LM nanoparticles after friction and chelation. The insets of SEM images display the changes in dimensional structures after the 1st and 50th rolling. (b and c) SEM images presenting the density of distributed Ga_2_O_3_-LM microspheres after the 50th rolling. (d) Sheet resistance of LM electrodes printed on gelatin, PDMS and PPH, respectively. (e) Photos of LM on gelatin, PDMS and PPH by roll-printing after scratching tests. Data are presented as mean ± SD (*n* = 3). (f) Images of F–F binding interface of LM electrodes. (g) Binding strength and bridging resistance of F–F connected LM electrodes with different thicknesses of PPH layer. Data are presented as mean ± SD (*n* = 3). (h) Binding strength and bridging resistance of F–F connected LM electrodes at different repeated times. Data are presented as mean ± SD (*n* = 3). (i) Images of E–E binding interface of LM electrodes. (j) Resistance change of F–E, E–E binding interfaces of LM electrodes as a function of tensile strain. (k) On-skin impedance tests of flexible LM electrodes (LM–PPH–PET), Cu–PPH hydrogel on flexible LM electrode (Cu–PPH gel on LM–PPH–PET), Cu–PPH hydrogel on E–F binding interface of LM electrodes (Cu–PPH gel on E–F interface) without/with stretching.

We introduced a roll-printing method of LM on PPH soft hydrogels, where the surface-oxidized LM microspheres (inside SEM image of Fig. 4a I) were squeezed into interconnected nanoparticles (inside SEM image of Fig. 4a II) under multiple frictions. Owing to the strong chelation between PPH and Ga^3+^, the hard and insoluble phytate shells were formed on LM nanoparticles, leading to the embedment of nanoparticles into soft hydrogel after 50 cycles of rolling. Moreover, the further oxidation of LM nanoparticles was inhibited due to the strong passivation layer.

One of the vital parameters for fabricating uniform and conductive LM films by roll-printing is the number of repeated times. As can be seen, separately distributed microspheres of Ga_2_O_3_-LM were formed on the PPH surface after the first rolling, which was unable to form conductive connections. After 10 cycles of roll-printing, the density of Ga_2_O_3_-LM particles increased, but the uniformity was still poor. Compared to the morphology of Ga_2_O_3_-LM after the first rolling, the SEM image of LM after 10 cycles of rolling displayed leakage of fluidic LM from the oxidized shell under friction force. The elemental distributions of Ga, In, C, P and O under different friction times were captured by an energy dispersive spectrometer (EDX) (Fig. S20). When the rolling time was increased to 50, homogeneously distributed Ga_2_O_3_-LM nanoparticles on PPH film were observed (Fig. 4b and c). The elemental distribution of Ga and In also indicated the evident changes after different rolling cycles.

To figure out the passivation effect of PPH on LM electrodes, similar printing steps were conducted on gelatin and PDMS. After 50 cycles of rolling, the LM on PPH was observed with much lower sheet resistance (0.33 Ω/sq) than that of LM on gelatin (1.0 Ω/sq) and PDMS (0.86 Ω/sq) (Fig. 4d). This is probably attributed to the passivation of PPH on LM, preventing the formation of surface oxides. Due to the phytate shell formed on the surface of LM, the scratching of LM on PPH shows nearly no residues on the glove, compared to the scratching tests of LM on gelatin and PDMS substrates (Fig. 4e). As a result, highly stretchable and conductive LM electrodes with a mechanically stable interface on PPH–PDMS can be achieved. The printed LM films on PPH–PDMS can be selectively patterned by laser processing (Fig. S21). Due to the high conductivity, the LM electrodes with a line width of 100 μm were able to light a red micro light emitting diode. Also, the LM nanoparticles on gelatin, PDMS and PPH after roll-printing enabled the patterning of micro-scale electrodes (∼50 μm in width) by using an UV nanosecond laser (Fig. S22a–c). The conductive LM electrodes on PPH, with varying line widths, exhibited lower resistance compared to those on gelatin and PDMS surfaces. When the line width of the patterned LM electrode was only 50 μm, the resistance was as low as 64.0 Ω, while the line resistances for LM on PDMS and gelatin were 126.2 and 231.7 Ω, respectively (Fig. S22d).

Another advantage of PPH-passivated LM electrodes is the strong binding adhesion of two printed LM electrodes on PPH–PDMS by a face-to-face attachment. During the rolling process, the LM nanoparticles were partially embedded into the hydrogel, and the exposed LM nanoparticles were protected by a passivation layer (Fig. S23). The patterned LM electrodes on PPH were ablated by a ultraviolet (UV) nanosecond laser system at a low fluence. Notably, the LM patterns were still conductive even after three cycles of ablation, indicating the penetration of LM in PPH film (Fig. S24). Obviously, the binding strength and bridging resistance between two surfaces of LM–PPH–PDMS were highly related to the thickness of LM under different rolling times. As the number of rolling cycles increased from 1 to 50, the binding strength declined from 430.8 to 132.7 kPa, while the bridging resistance dropped significantly from 1.2 MΩ to 0.77 Ω. (Fig. S25). However, further increasing rolling cycles to 100 led to the growth of bridging resistance due to the surface oxidation. Also, the thickness of the PPH layer also showed a slight effect on the adhesion strength between F–F-connected LM electrodes after 50 cycles of rolling (Fig. 4f and g). Due to the partial penetration of LM nanoparticles into the PPH film, the adhesion strength was significantly influenced by the interlocking structure between the two LM surfaces, which possessed different nanoparticle densities [49]. An excessive accumulation of LM nanoparticles on the PPH surface weakened the binding strength by hindering the formation of a sufficient interlocking structure. Meanwhile, the ultrathin PPH coated at a speed of 5000 r/min was difficult to protect the surface LM from oxidation by passivation, which presented evidently increased bridging resistance (∼10 Ω/sq).

Interestingly, the binding strength between the surfaces of LM–PPH–PET was enhanced by repeat attachments (Fig. 4h). As the interfacial separation and attachment was repeated for five cycles, the binding strength was increased from 181.3 to 216.2 kPa. Moreover, even after 20 cycles of repeat tests, the binding strength could still reach 183.9 kPa. During this process, a slight increase of bridging resistance from 2.1 to 4 Ω/sq was observed. Such shear-reinforced adhesion strength of LM surfaces was attributed to the interlocking of the surface-passivated LM nanoparticles under pressure force. Due to the hard surface of the passivation layer, the interlocked structure could be rebuilt for several repeats of separations and contacts. Overall, the strong mechanical and electrical connections of PPH-passivated LM surfaces enable another way of E–E binding interface for bioelectronics (Fig. 4i). The E–E binding interface of LM electrodes fabricated by roll-printing was observed with a high intrinsic stretchability of 120%. The F–E and E–E binding interfaces of LM-based electrodes were observed with almost no resistance change below a strain of 19% and 12.8%, respectively (Fig. 4j). Even when the E–E binding interface of LM electrodes was stretched to 120%, the bridging resistance (∼222 Ω) was still acceptable for the detection of high-fidelity electrophysiological signals. To validate the performance of bidirectionally bound interfaces for electrophysiological signal recording, the contact impedances of LM–PPH–PET dry electrodes, Cu-PPH hydrogels on LM–PPH–PET and E–F binding interface of LM electrodes on skin were measured at a frequency range of 100 Hz–100 kHz (Fig. 4k). It can be seen that the internally chelated Cu–PPH hydrogel on the LM–PPH electrode and E–F-bridged LM electrode showed similar impedance (∼30 kΩ) at 100 Hz, which is much lower than that of the dry electrode (∼121.9 kΩ). This indicates that the soft and wet biointerfaces are better for high-fidelity electrophysiological signal recording. Furthermore, the bidirectional interface of soft Cu–PPH on E–F-bridged LM electrodes was observed with stable contact impedance on skin, even under stretching.

### Chelation-induced bidirectional biointerfaces

**Figure 5.**
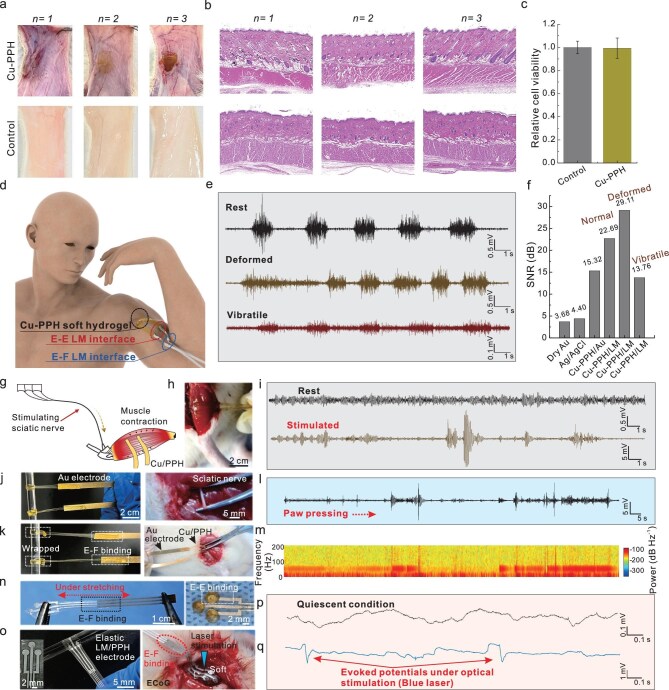
Bidirectional electrical interface or electrophysiological recording. (a and b) Histological analysis of subcutaneous tissue of rats in control group and Cu–PPH hydrogel group after 7 days of implantation. (c) Cytotoxic results of control group and Cu–PPH hydrogel group. Data are presented as mean ± SD (*n* = 3). (d) Schematic of soft Cu–PPH hydrogels bridged with E–F binding interface of LM electrodes for EMG detection. (e) EMG signals of Cu–PPH/LM electrical interface on the arm under calm state, deformed state of skin, and vibratile state of muscle by holding a weight of 2 kg. (f) SNR calculated by the EMG signals of dry Au, commercial Ag/AgCl gel, Cu–PPH/Au and Cu–PPH/LM electrodes. (g and h) Schematic and photo of Cu–PPH hydrogels attached onto the exposed muscle of a rat for EMG recording. (i) The EMG signals of a rat collected using soft Cu–PPH hydrogels as contact electrode and flexible Au–PPH–PET as connection electrode at states of rest and stimulation. (j and k) Photos of the Cu–PPH hydrogel-based sleeves wrapped around a glass rod and the sciatic nerve electrically connected by the Au–PPH–PET electrodes. (l) Electrical signals of the sciatic nerve under stimulation recorded by Cu–PPH hydrogel-based sleeves by paw pressing. (m) Spectrograms of the recorded neural signals under stimulations. (n) Photos of Cu–PPH hydrogels patterned into circle shapes with a diameter of 3 mm connected by the E–F binding interface of LM electrodes. (o) Photo (left) of the stretchable LM–PPH–PDMS electrode applied as the elastic binding interface (E) for Cu–PPH hydrogel (E) that was wrapped around a glass rod. Photo (right) of the patterned Cu–PPH hydrogels for ECoG signal recording connected with the E–F binding interface of LM electrodes. (p and q) ECoG signals recorded by the soft hydrogel electrode under the normal condition and optical stimulation of a blue laser.

Benefiting from the strong chelation ability of PA with metals, the internally chelated conductive Cu–PPH hydrogels bridged with surface-chelated Au and LM electrodes were successfully fabricated for electrophysiological signal recording. Hydrogels, the Young’s modulus of which is much lower than that of elastomers, are often applied as the contact electrodes with tissue for high-fidelity electrophysiological signal recording. To evaluate the advantages of this hybrid electrical interface, which features a soft Cu–PPH hydrogel and an Au–PPH electrode, electromyographic (EMG) signals were recorded and compared with those obtained using a dry Au electrode and a commercial wet gel. In this system, copper ions exist in a chelated and stabilized state. Combined with previous studies, it has been shown that such coordination structures exhibit acceptable biocompatibility within a certain concentration range [52]. The hematoxylin & eosin (H&E) staining results revealed the tissue morphology with no evident inflammation response, and the CCK-8 essay revealed that the relative cell viability of the Cu–PPH group is about 99%. Both confirmed the high biocompatibility of Cu–PPH hydrogels (Fig. 5a–c). It is evident that the dry Au electrode, when naturally attached to skin and immobilized by a polyurethane tape, struggles to capture EMG signals due to the invisible gap between the electrode and the skin. Under an external pressing force, the Au electrode was able to record the EMG signals, yet significant mechanical interference could be also observed. In contrast, the EMG signals recorded using the bidirectional electrical interface of Cu–PPH hydrogels and flexible Au electrodes display a clear baseline and higher SNR value than that of commercial gel using button electrodes (Fig. S26). To demonstrate the reliability of this bidirectional binding interface for practical bioelectronic applications, EMG signals were collected on the upper arm of an adult at different states (Fig. 5d). Specifically, the Cu–PPH hydrogel was applied as the adhesive contact electrode. The E–E and E–F bridged LM electrodes were used as the stretchable and interconnection electrodes, respectively. The adhesion of this soft hydrogel was captured by attaching on the skin under stretching (Supplementary Video 5). To check the dynamic conformance of Cu–PPH electrodes with skin, the EMG signals were detected and compared when the skin was in flat and deformed states. The SNR values were over 20 dB and the deformed state showed higher SNR due to the high conformability of Cu–PPH hydrogel and skin (Fig. 5e and f). EMG signals were recorded under high-frequency muscle vibrations while holding a 2 kg weight, resulting in an SNR of just 13.76 dB. This reduction was due to interference from noise signals originating from multiple muscle groups, which weakened the biceps’ electrical signals during the lift. Notably, the ultrasoft Cu–PPH hydrogel and LM electrode interface provided superior SNR compared to dry Au electrodes and commercial gel electrodes, ensuring stable measurements even under skin deformations.

In addition, the soft Cu–PPH hydrogels connected by Au–PPH–PET electrodes were also applied for acquisition of electrical signals on muscles of rats by stimulating the sciatic nerve (Fig. 5g and h). In the rest state, the electrical signals of muscle were very weak and the amplitude was around 0.5 mV. Under stimulation, the contraction of muscle at the lower limb resulted in a dramatic increase of electrical signals, the amplitude of which was over 5 mV (Fig. 5i). Furthermore, the soft Cu–PPH hydrogel was also applied as a nerve sleeve for neural signal recording. The hydrogel strips conformally wrapped around the sciatic nerve using the self-healing property. The other end of the hydrogel was connected with an Au–PPH–PET electrode by chelation (Fig. 5j). Both the sleeves and chelated interfaces were mechanically stable even under stretching (Fig. 5k). Evident bursts of spikes of amplitude and frequency domain features, under sensory stimuli, such as paw pressing, were clearly captured by the soft hydrogel-based sleeves (Fig. 5l and m). The obtained amplitude of signal under stimuli was nearly over 10 mV, which indicates the low contact impedance between the soft Cu–PPH hydrogel-based sleeves and the sciatic nerve.

Additionally, we also employed the soft hydrogel electrode (diameter: 3 mm) to measure the electrocorticogram (ECoG) signals in the brain of rats. The bidirectional binding interface for ECoG recording consisted of an E–E interface between the circular hydrogel electrode and the patterned LM–PPH–PDMS layer (thickness: ∼22 μm), as well as an E–F interface between the patterned LM–PPH–PDMS and LM–PPH–PET layer (thickness: ∼53 μm) for circuit connections. (Fig. 5n, Supplementary Video 6). The thin LM–PPH–PDMS was highly stretchable and mechanically compatible with the soft hydrogels on brain tissues (Fig. 5o). The small Cu–PPH hydrogel electrodes were gently and seamlessly attached onto the brain for recording the ECoG signals at normal and light evoked states (Fig. 5p and q). Upon blue laser (wavelength: 450 nm) stimulation, prominent negative deflections in the cortical potential were observed, corresponding to evoked action potentials. These transient spikes suggest rapid neuronal depolarization and firing synchronized with the onset of optical stimulation. Overall, these bidirectional binding interfaces with a gradient modulus design significantly improved the practicality of hydrogels on fragile tissues while ensuring stable electrophysiological signal recording with high SNR values.

## CONCLUSIONS

In this work, a bidirectional electrical interface of hydrogel and metal electrodes is developed through coordination interactions, effectively bridging soft skin/tissue and data collection circuits. This interface leverages a dual-mode chelation mechanism, consisting of both internal and surface chelation, to optimize the cross-linking structure of this hydrogel and reinforce the metal–hydrogel binding interface. Internally, strong chelation competes with esterification, resulting in a tissue-like softness with an ultra-low modulus of approximately 339.9 Pa, ensuring seamless attachment to biological surfaces. Externally, the hydrogel passivates diversity of metal surfaces, i.e. metal foils, Au and LM films, facilitating the formation of interlocked structures on bonded metal surfaces. This surface chelation enabled a high binding strength of ∼1.95 MPa between the Cu foils with a thickness of 100 μm without compromising electrical conductivity. As a result, a hybrid electrical interface between soft hydrogels and flexible or elastic metal electrodes enables a bidirectional connection to skin/tissue and circuits. This allows for high-SNR electrophysiological recordings from the skin, neural surfaces and the brain, while ensuring reliable performance under mechanical disturbances from biological activities.

## METHODS

### Fabrication of soft hydrogels by internal chelation

The precursor materials for the fabrication of metal-chelated hydrogels include PVA (87%–89% hydrolyzed, Aladdin), PA (50% wt in water, Macklin), honey, Cu nanoparticles (50 nm in diameter, Leber Metal Material Technology Co., LTD) and LM (DG-16, Dingguan Metal Technology Co., Ltd). First, 10% PVA solution was prepared by dissolving PVA particles in DI water, then 0.5%, 1% and 2% Cu nanoparticles and LM, accounting for the mass of PVA solution (10 g), were dispersed in DI water by tubular ultrasonication for 10 min. The dispersed nanoparticles of Cu and Ga_2_O_3_ with different contents were collected via centrifugation at 5000 r/min for 5 min. Subsequently, the obtained metal nanoparticles in different centrifuge tubes were mixed with 10 g of PVA solution, 5 g of PA solution and 4 g of honey under 30 min of stirring. The obtained mixture solutions were then heated inside a hot water bath (80°C) to facilitate the cross-linking and chelating processes of polymers and metal ions until the solutions looked transparent and light brown. After cooling for 30 min, the chelated and chemically cross-linked polymer solutions were poured into PDMS molds for physical cross-linking and solidification for at least three cycles of freeze-thawing treatments. The pristine PPH hydrogels were fabricated using the same steps without adding any metal nanoparticles.

### Fabrication of F–F bridged metal foils by surface chelation

Similarly, hydrogels used for interfacial chelation with metal foils (i.e. Cu, Mo, Fe, Ni, Al, Ag) were also prepared using the above steps without adding metal nanoparticles. After cooling down, the partially cross-linked PPH hydrogel solution was spin-coated onto the surface of these metals. The thickness of the PPH hydrogels formed on metals can be controlled by the speed of spin coating. At the same time, the mass ratio of PVA and PA (1:1, 2:1, 4:1, 8:1 and 16:1) was also adjusted to study the effect of PA content on binding strength of chelated interface between metals. The mass ratio of PVA to honey was controlled to 5:2. After coating, the metal foils with ultrathin hydrogel were attached to another surface of the same metal and the combined area was hot pressed at room temperature for 2 min. Finally, the combined samples were placed at room temperature for adequate interfacial chelation.

### Statistics and reproducibility

All the experimental data were statistically analysed and the results are expressed as mean ± SD (*n* ≥ 3). Excel, Origin 2018, MATLAB R2023a and Python software were used for data analysis. The raw data of surface EMG, *in vivo* EMG and neural signal were processed with a bandpass filter (6–400 Hz). The SNR of electrophysiological signals was calculated by normalizing the raw data of surface EMG signals.

## ETHICAL STATEMENT

Male Sprague-Dawley (SD) rats (200±20 g) used for all the electrophysiological detections were obtained from GemPharmatech Co., Ltd. The study protocol associated with wearable sensors was approved by the ethical committee of College of Biomedical Engineering & Instrument Science, Zhejiang University ([2023]-7). It was stated that ethical experiments on human subjects were conducted with the consent of the subjects. All animal procedures were approved by the Animal Care and Use Committee of the Second Affiliated Hospital of Zhejiang University School of Medicine (NoZJU20240573).

## Supplementary Material

nwaf399_Supplemental_Files
