# Supplementary material for: Metal–hydrogel chelation interfaces for ultrasoft and bidirectional bioelectronics
Source: Natl Sci Rev. 2025 Sep 18;12(11):nwaf399. doi: 10.1093/nsr/nwaf399 (PMC12618001; doi:10.1093/nsr/nwaf399)
Supplement: nwaf399_Supplemental_Files [file nwaf399_supplemental_files.zip › Supplementary data.pdf]

Supporting information

Metal-hydrogel chelation interfaces for ultrasoft and bidirectional bioelectronics

Yuyao Lu[1,*], Ziguan Jin[1], Yihui Jian[1], Depeng Kong[1], Hao Zhou[1], Yuhong Xu[1], Ruijue Cao[2], Zhuoheng Xia[3], Fan Yang[2], Qianglong Wu[4], Yang Gao[4], Aoran Cui[5], Shikuan Yang[5], Nenggan Zheng[6], Junhyuk Bang[7], Geng Yang[1,8,*], Seung Hwan Ko[7], Huayong Yang[1] and Kaichen Xu[1,*]

[1]State Key Laboratory of Fluid Power and Mechatronic Systems, School of Mechanical Engineering, Zhejiang University, Hangzhou 310027, China;

[2]Center for Plastic & Reconstructive Surgery, Department of Stomatology, Zhejiang Provincial People's Hospital, Affiliated People's Hospital, Hangzhou Medical College, Hangzhou 310014, China;

[3]School of Stomatology, Zhejiang Chinese Medical University, Hangzhou 310053, China;

[4]Center for X-mechanics, Department of Engineering Mechanics, Zhejiang University, Hangzhou 310027, China;

[5]Institute for Composites Science Innovation, School of Materials Science and Engineering, Zhejiang University, Hangzhou 310027, China;

[6]State Key Laboratory of Brain-Machine Intelligence and the Qiushi Academy for Advanced Studies, Zhejiang University, Hangzhou 310027, China;

[7]Department of Mechanical Engineering, Seoul National University, Seoul, Republic of Korea;

[8]Zhejiang Key Laboratory of Intelligent Robot for Operation and Maintenance, Hangzhou 310000, China;

[*]**Corresponding authors.** E-mails: lu_yy@zju.edu.cn; yanggeng@zju.edu.cn; xukc@zju.edu.cn

Methods

Fabrication of E-E and E-F bridged Au and LM electrodes by surface chelation

The PPH-PET and PPH-PDMS-PET substrates for Au deposition and LM printing were initially prepared by spin-coating uncured PDMS precursor (ratio of PDMS and curing agent is 20:1, Sylgard 1844, Dow corning) on PET substrates. After baking at 90 ℃ for 30 min, the partially crosslinked PPH hydrogel solution (ratio of PVA, PA and honey is 10:5:4) was spin-coated onto the surface of PET and PDMS-PET. For flexible Au-PPH-PET and stretchable Au-PPH-PDMS samples, the thickness of Au (20 nm, 40 nm, 60 nm) was controlled by deposition time of a thermal evaporation system (VZZ-300S, VNANO). The LM electrodes were obtained by roll-printing the mixture of liquid metal (ratio of Ga and In of 3:1) and copper nanoparticles (50 nm, 99.9%) on the PPH surface for different cycles (1, 10, 50, and 100, respectively). A critical step is that 2% Cu nanoparticles (diameter: ~50 nm) were mixed with LM using a high-speed mixer to decrease the surface tension before roll printing. The mixing ratio of liquid metal and copper is 98:2 and the mixing process was conducted by a vortex mixer (AR100, Thinky) at 2000 RPM For the roll-printing step, the pre-printing of the LM on oiled paper was accomplished until the Ga_2O_3-LM microspheres were uniformly distributed on a rubber roller. Then, surface-passivated LM films were obtained by multiple roll printing of Ga_2O_3-LM on PPH-PDMS.

Laser patterning of Cu-PPH hydrogel, Au-PPH and LM-PPH for electrophysiological sensors

The Cu-PPH hydrogels used as soft contact electrodes for EMG and ECoG detections were designed and cut into long strips (length: 3 cm, width: 5 mm) and small discs (diameter: 3 mm) by an infrared CO_2 laser system (VLS3.60, UNIVERSAL, USA). To ensure biocompatibility, these hydrogels were dipped into PBS solution for several times to remove the extra acids until pH value reached 6-7. The surface of swollen hydrogels was wiped by non-woven fabrics before use. The Au-PPH and LM-PPH based connection electrode arrays (1×6, length: 3 cm, width: 0.6 mm) on PET (F-F binding) and PDMS-PET (E-F, E-E binding) substrates were designed and patterned by an ultraviolet nanosecond laser system (355 nm, FOTIA, INNO Laser, China).

Characterizations

The rheological property of PPH, Cu-PPH and Ga-PPH hydrogels were analyzed by a discovery hybrid rheometer (DHA, Kinexus Lab+, NETZSCH). The surface morphology of PPH treated Cu and LM nanoparticles was captured by a scanning electron microscope (SEM, G300, ZEISS). The surface roughness and height difference between Cu and PPH treated Cu were analyzed by an atomic force microscope (AFM, MFP-3D BIO, OXFORD INSTRUMENTS). The elemental composition and chemical state analysis of the pure Cu and PPH treated Cu were conducted by an x-ray photoelectron spectroscopy (XPS, EscaLab 250Xi, Thermo Fisher Scientific). The tensile performance of PPH, Cu-PPH and Ga-PPH was tested by a material tensile apparatus (34sc-05, INSTRON). The binding strength of PPH hydrogel as an adhesive and chelation layer between metal foils, Au nanoparticles and LM was measured by lap shear tests using a tensile apparatus (ZQ990B, ZHIQU). The resistance change of E-F and E-E combined electrodes under tensile strain was recorded by a multimeter (34661A, Keysight). The electrophysiological signals including EMG, neural signal recording and ECoG were detected by a self-designed printed circuit board (PCB) circuit with a sampling rate of 2000 Hz and wireless data transmission function via Bluetooth. The skin/electrode impedance test and electrochemical impedance spectroscopy (EIS) were conducted by a benchtop LCR meter (E4980AL, KEYSIGHT), respectively.

Agar disk diffusion assay. *In vitro* antibacterial experiment was carried out by an agar disk diffusion assay. Briefly, the PPH hydrogel, Cu-PPH and Ga-PPH hydrogels were cut into 8-mm-diameter pieces. The agar mixture was melted in an autoclave pot and then poured onto petri dishes (90-mm diameter) before it was cooled down to 60 ℃. 100 μL of bacterial suspensions of Methicillin-resistant Staphylococcus aureus (MRSA, ATCC33591, 2.5×10^6 CFU/mL) were uniformly swabbed onto the Mueller–Hinton agar plates. All the samples were incubated at 37 ℃ for 12 h and 2 weeks. After the incubation, the samples were taken out for photographing.

Animals and treatment. Male Sprague-Dawley (SD) rats (200±20 g) used for all the electrophysiological detections were obtained from GemPharmatech Co., Ltd. The study protocol associated with wearable sensors was approved by the ethical committee of College of Biomedical Engineering & Instrument Science, Zhejiang University ([2023]-7). It was stated that ethical

experiments on human subjects were conducted with the consent of the subjects. All animal procedures were approved by the Animal Care and Use Committee of the Second Affiliated Hospital of Zhejiang University School of Medicine (NoZJU20240573).

Molecular dynamics (MD) calculation

First of all, a polyvinyl alcohol structure containing 10 repeating units was constructed, FFMM94 force field was applied for structural optimization, and Open-source Radio Clock and Alarm (ORCA) was used to calculate the Generalized Amber Force Field (GAFF) parameters of polyvinyl alcohol. Similarly, use ORCA to calculate the GAFF force field parameters of glucose and PA. Then, two systems were constructed separately and simulations were conducted by a software named as GROningen MAchine for Chemical Simulation (GROMACS). System 1 contained PVA, water, glucose and Cu^{2+} with a molar ratio of 1: 22.00: 0.978: 0.0693, the numbers of molecules placed in this system are 35, 7700, 343, 50, respectively. System 2 contained PVA, water, glucose, PA and Cu^{2+} with a molar ratio of 1: 28.12: 0.978: 0.167: 0.0693, the numbers of molecules are 35, 9835, 343, 58, 50, respectively.

Specifically, in each system, a box of about 10*10*10 nm^3 was constructed, the above-mentioned molecules were randomly placed in the box, the box was filled with water solvent, Cl and Na ions were used to balance the charge in the boxes. Gromacs was applied to perform energy minimization, NVT balance, NPT balance and finished product simulation for 100 ns. NVT and NPT were mainly used to constrain the number of atoms (n), temperature (t), volume (v) and pressure (p) of the two systems. The temperature and pressure applied in the two simulation systems were 353.15 K (i.e. 80 degrees) and ambient pressure, respectively.

The finished simulation used the leapfrog algorithm to integrate Newton's equations of motion, and the integration time step is 2 fs. The finished simulation used the V-rescale temperature coupling method and the Parrinello-Rahman pressure coupling method. The nearest neighbor search conducted using the Verlet method. The Coulomb and van der Waals interaction cutoff radius was 1.4 nm, and the long-range electrostatic interaction was calculated by the Particle-Mesh Ewald (PME) method. Long-range dispersion correction is used for energy and pressure.

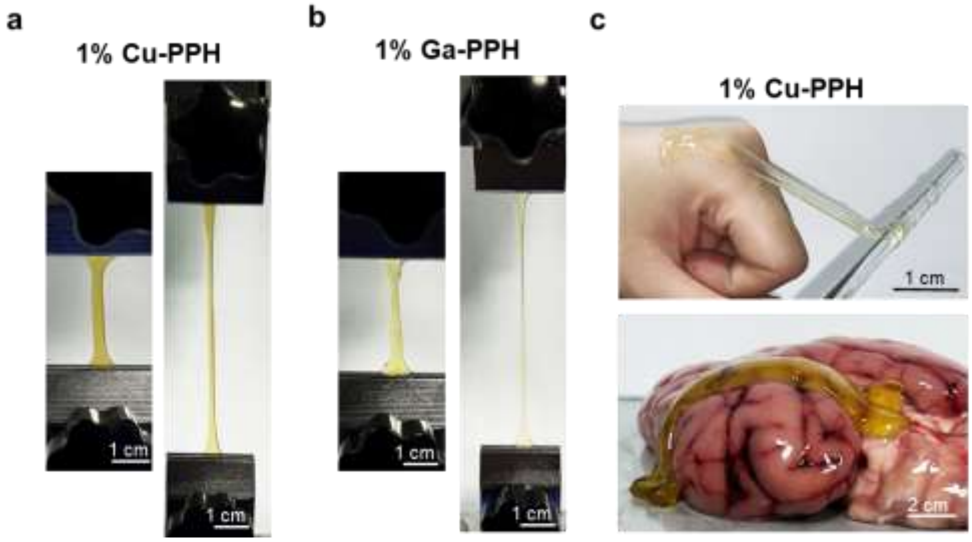

Supplementary Fig. 1: Photo images of internally chelated soft hydrogels. a, b, Photos of stretched Cu-PPH and Ga-PPH hydrogels. **c,** Photos of soft Cu-PPH hydrogel attached on skin and brain tissue.

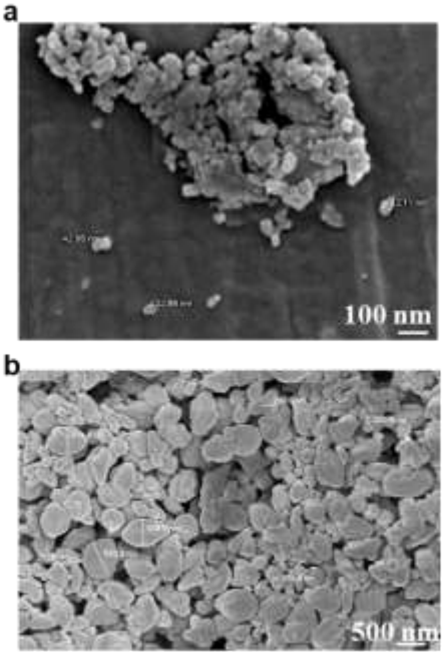

Supplementary Fig. 2: Scanning electron microscope (SEM) images of the (a) Cu nanoparticles and (b) Ga_2O_3-LM microspheres.

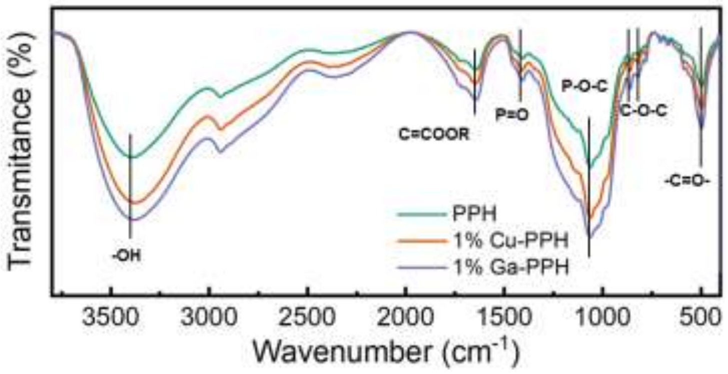

Supplementary Fig. 3: Infrared (IR) spectra of PPH hydrogels, 1% Cu-PPH hydrogels and 1% Ga-PPH hydrogels.

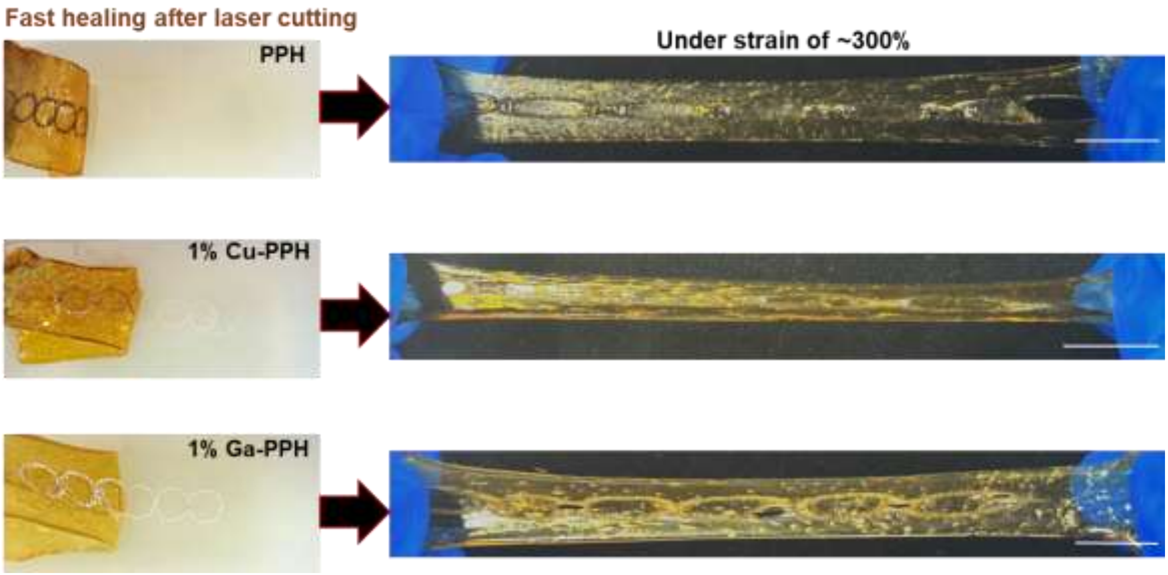

Supplementary Fig. 4: Self-healing property of PPH, 1% Cu-PPH and 1% Ga-PPH hydrogels under laser cutting. The images presented the stretchability of these hydrogels with fast-healing performances under laser cutting. Scale bar: 1 cm.

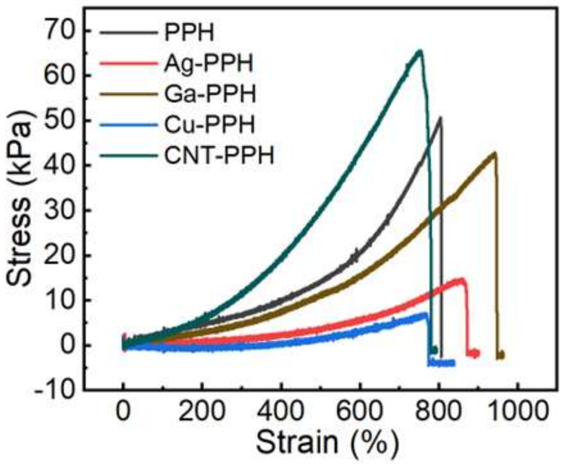

Supplementary Fig. 5: Tensile performances of different metal-chelated hydrogels and the CNTs-doped hydrogel. Metallic nanomaterials include Ag nanowires, Cu nanoparticles and Ga_2O_3-LM microspheres.

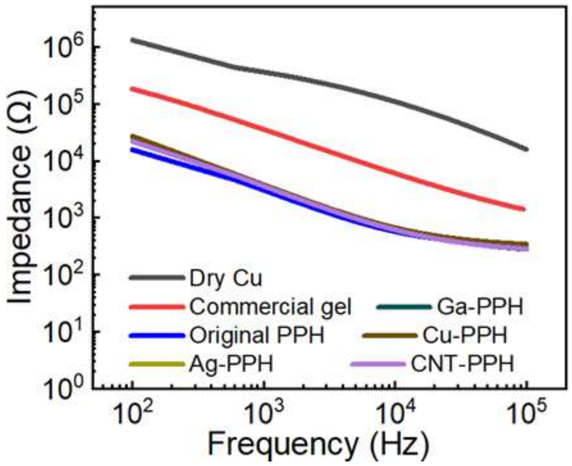

Supplementary Fig. 6: On-skin contact impedances of dry Cu, commercial gel, PPH hydrogel, CNTs-doped PPH hydrogel as well as metal-chelated PPH hydrogels. Metallic nanomaterials include Ag nanowires, Cu nanoparticles and Ga_2O_3-LM microspheres.

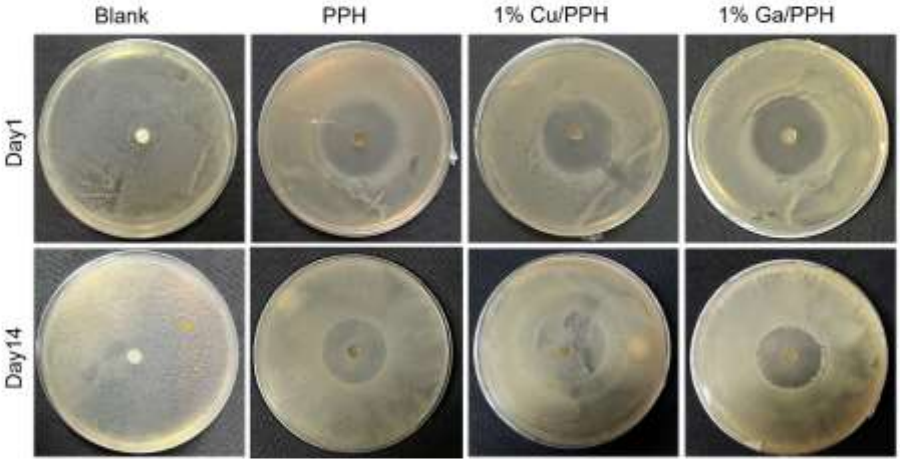

Supplementary Fig. 7: Anti-bacterial performances of blank filter paper, PPH, 1% Cu-PPH and 1% Ga-PPH that were cut into circle pieces with a diameter of 6 mm. The inhibition zone pictures in the first and second rows were recorded on day 1 and day 14, respectively.

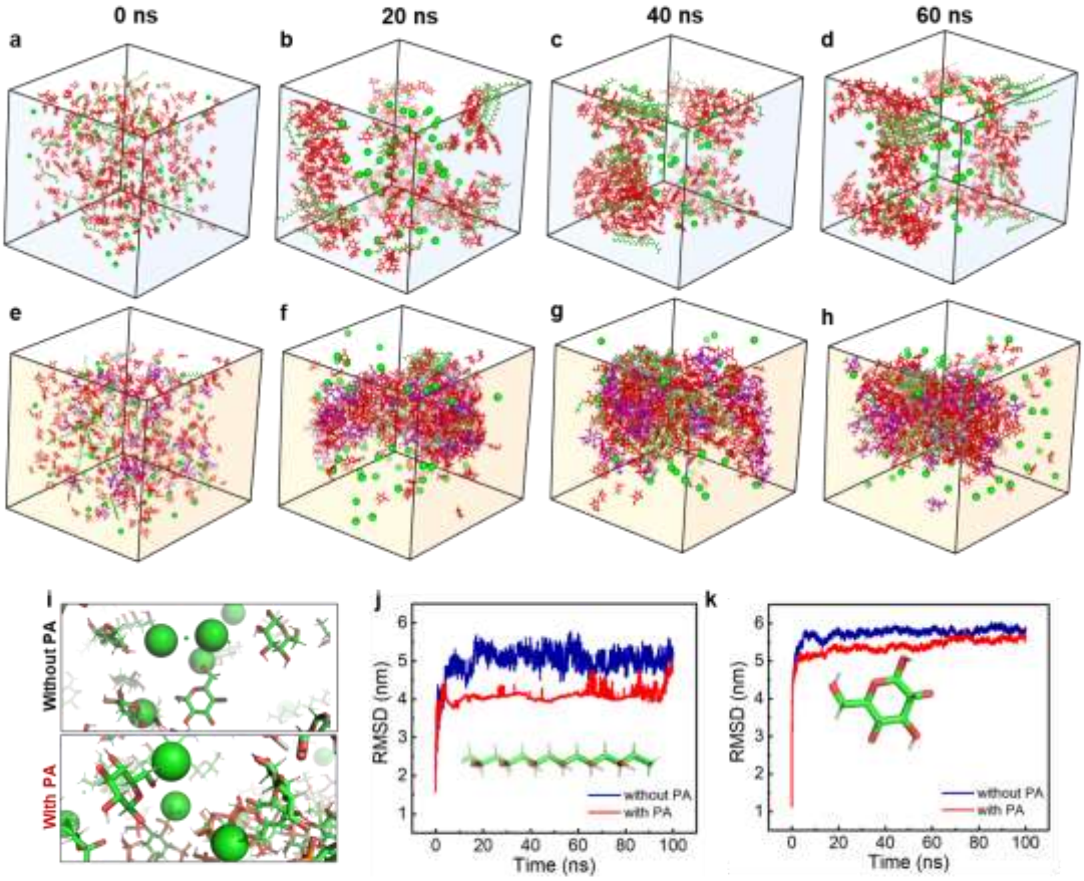

Supplementary Fig. 8: Molecular dynamics (MD) simulation results of Cu-PPH hydrogels wo/w phytic acid (PA). Snapshots of Cu-PPH composite hydrogel without chelation agent of PA at 0 ns (**a**), 20 ns (**b**), 40 ns (**c**), 60 ns (**d**), respectively. Snapshots of Cu chelated PPH hydrogel with PA at 0 ns (**e**), 20 ns (**f**), 40 ns (**g**), 60 ns (**h**), respectively. **i,** Magnified snapshots of different components distributed around Cu^{2+} in the presence and absence of PA. **j,** Radial distribution function (RDF) g(r) of Cu^{2+} and different molecules for the PPH hydrogels in the presence and absence of PA. **k,** Root mean square deviation (RMSD) results of PVA in the presence and absence of PA within 100 ns.

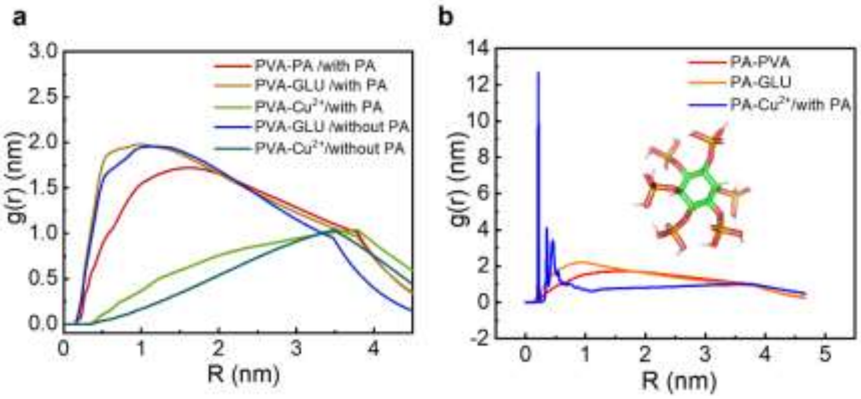

Supplementary Fig. 9: Radial distribution function (RDF) results of PPH hydrogels in the presence and absence of PA. a, g(r) of PVA and different molecules**. b,** g(r) of PA and different molecules.

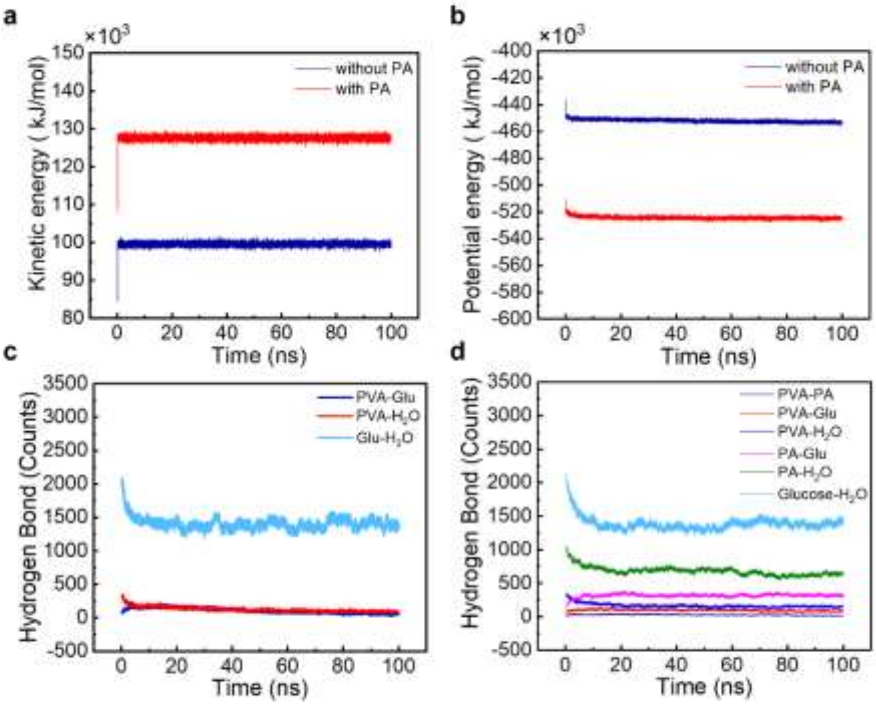

Supplementary Fig. 10: Molecular dynamics (MD) calculation results of the two hydrogel systems without and with PA. a, b, Kinetic energy and potential energy of the two hydrogel systems simulated in 100 ns. **c, d**, Number of hydrogen bonds formed in the two hydrogel systems.

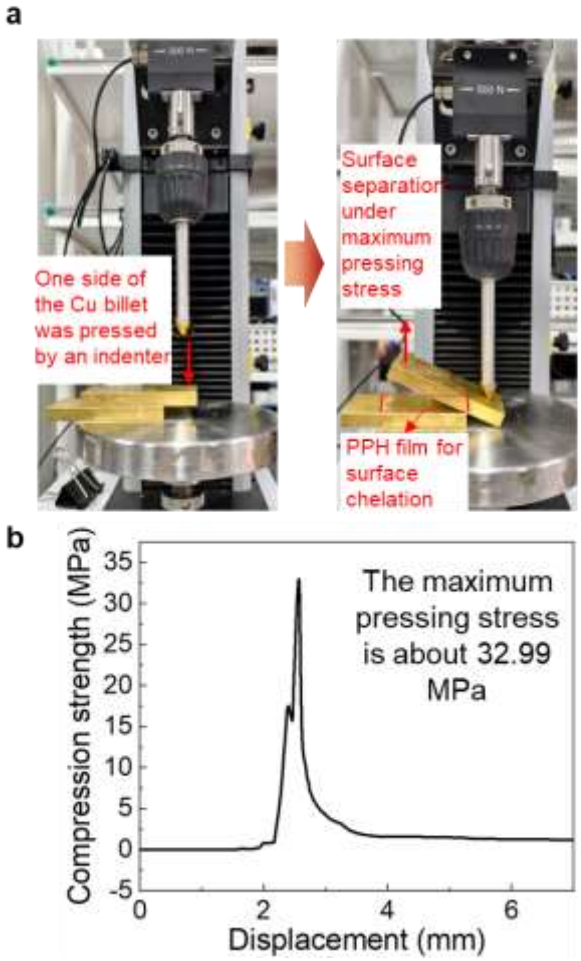

Supplementary Fig. 11: Adhesion strength of two Cu billets under surface chelation a, Photos of the compression test. **b,** The maximum pressing stress measured for separating two Cu billets under surface chelation.

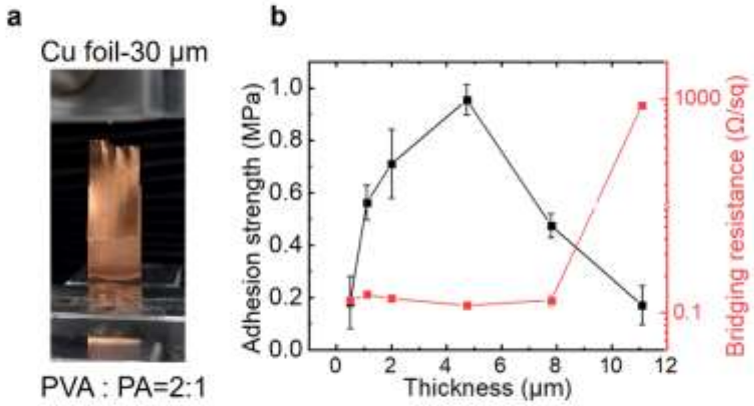

Supplementary Fig. 12: Adhesion strength of two pieces of Cu foils (30 µm) with different thicknesses of PPH adhesive layer. a, Photo of a fractured Cu foil strongly connected to another Cu foil via surface chelation by PPH hydrogel. **b,** Adhesion strength and bridging resistance between the two Cu foils by surface chelation. Data were presented as means ±SD, n=3.

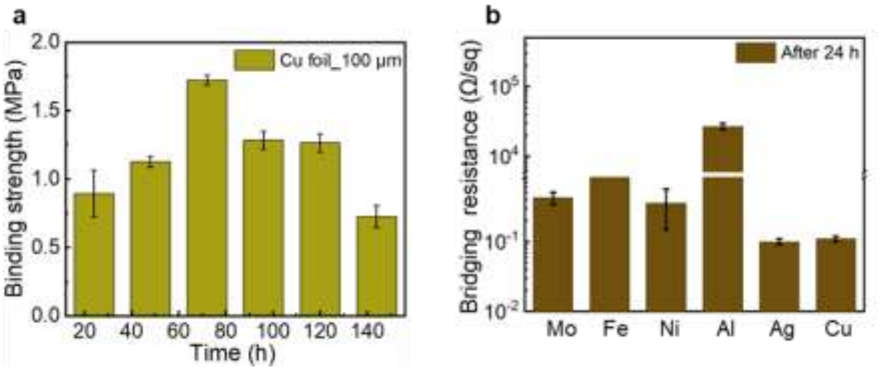

Supplementary Fig. 13: Long-time binding strength and bridging resistance of different metal foils using the PPH as the surface chelation agent and adhesion layer. a, Binding strength of two pieces of Cu foils investigated by lap shear tests under different chelation times. **b,** The bridging resistance of different metal foils after 24 h of surface chelation. Data were presented as means ±SD, n=3.

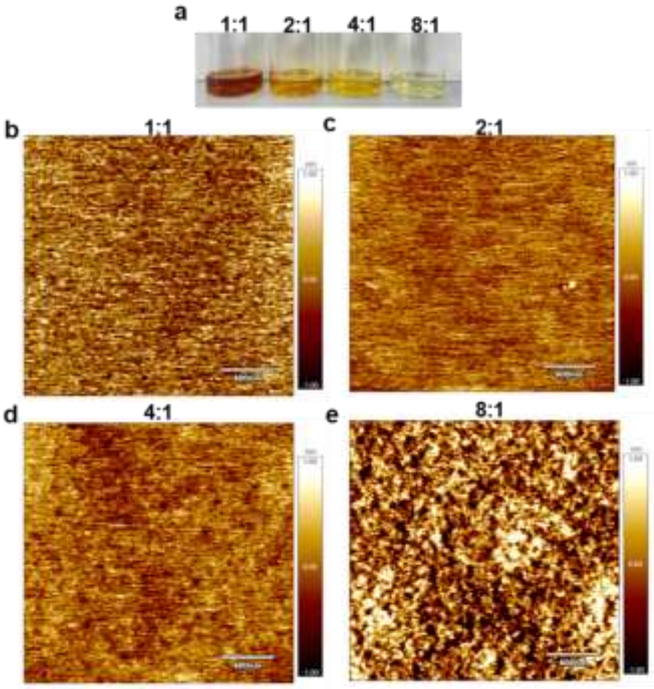

Supplementary Fig. 14: Atomic force microscopy (AFM) images of PPH hydrogels with different ratios of PVA and PA. a, Photo of partially crosslinked PPH solutions with different ratios of PVA and PA. **b, c, d, e,** Phase maps of PPH hydrogels with different ratios of PVA and PA.

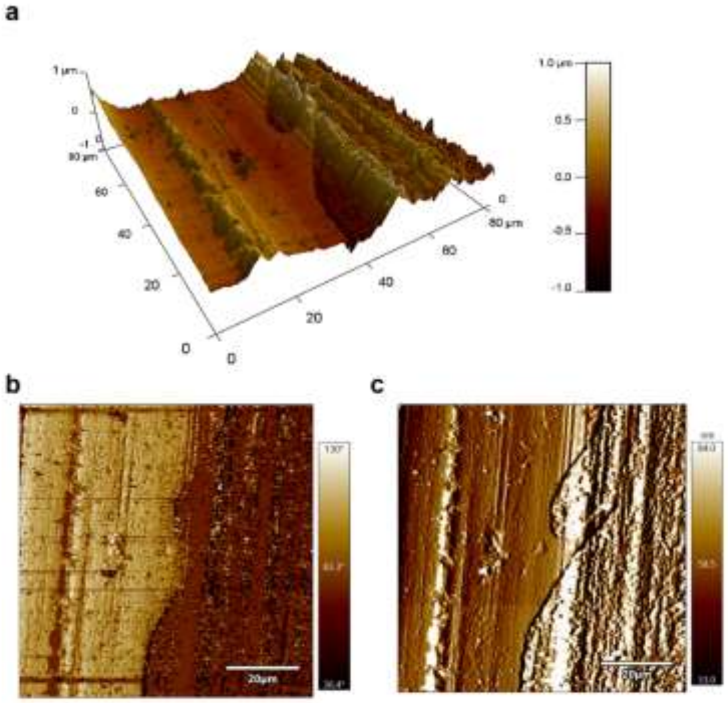

Supplementary Fig. 15: Surface topography and roughness maps of Cu foil before and after surface treatments by a thin layer of PPH. a, 3D mapping result of the pristine Cu (left area) and PPH-treated Cu (right area). **b, c,** Phase and amplitude maps of Cu foils before and after treatment by a thin layer of PPH.

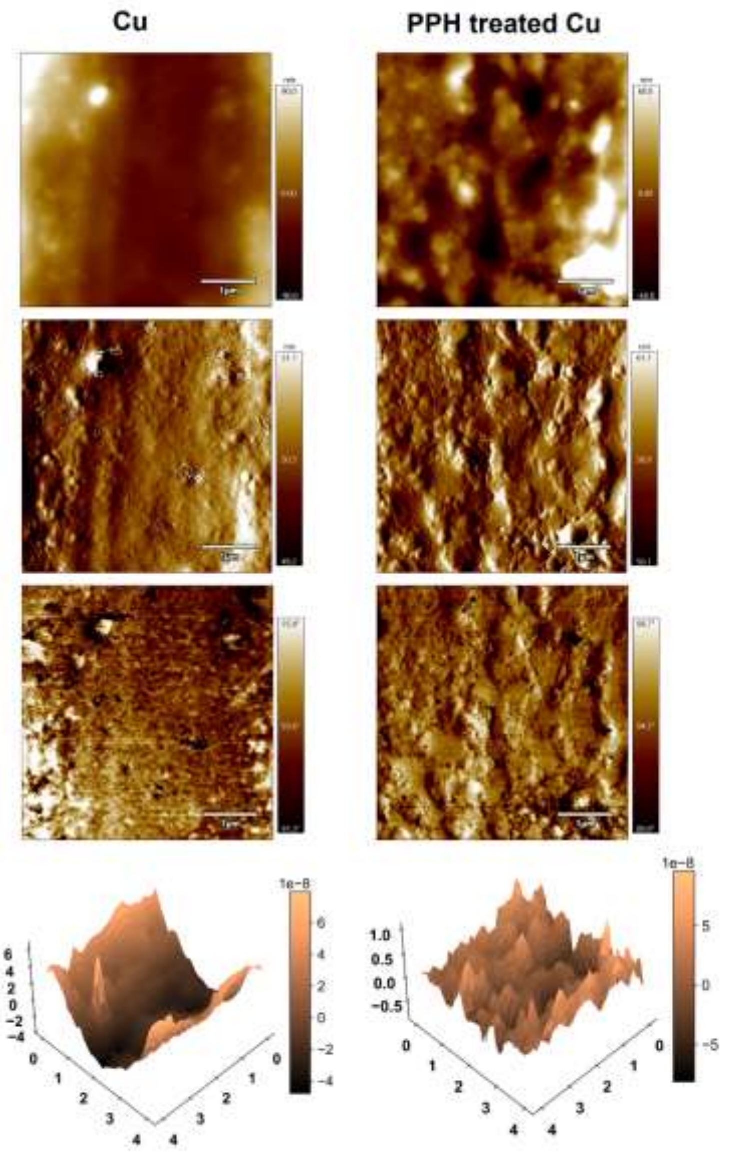

Supplementary Fig. 16: Surface topography, phase and amplitude maps of Cu foils before and after treatments by a thin layer of PPH.

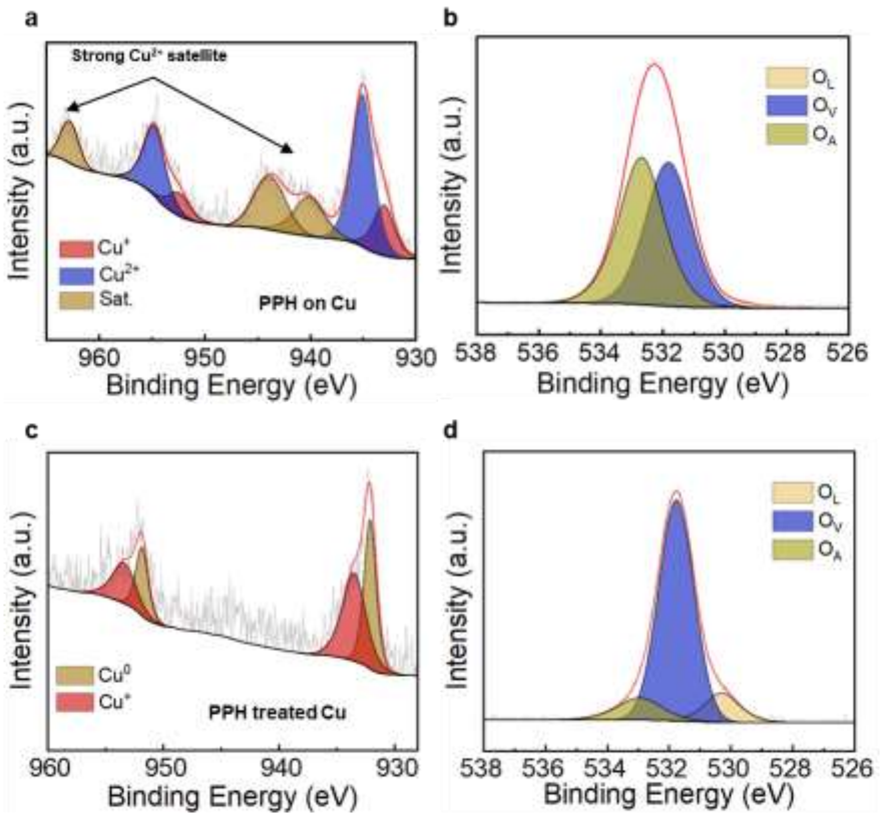

Supplementary Fig. 17: The x-ray photoelectron spectroscopy (XPS) results of Cu and O on surfaces of PPH coated Cu and PPH treated Cu after removing PPH. a, b, XPS spectra of Cu and O on PPH coated Cu. **c, d,** XPS spectra of Cu and O on PPH treated Cu.

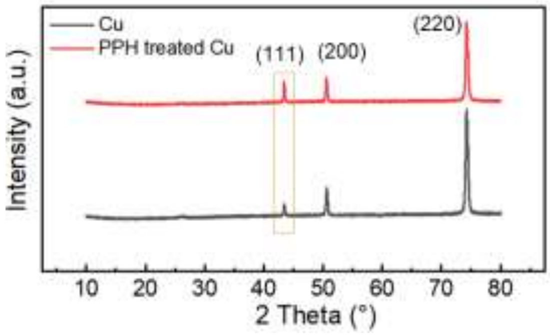

Supplementary Fig. 18: X-ray Diffraction (XRD) results of pristine Cu and PPH treated Cu.

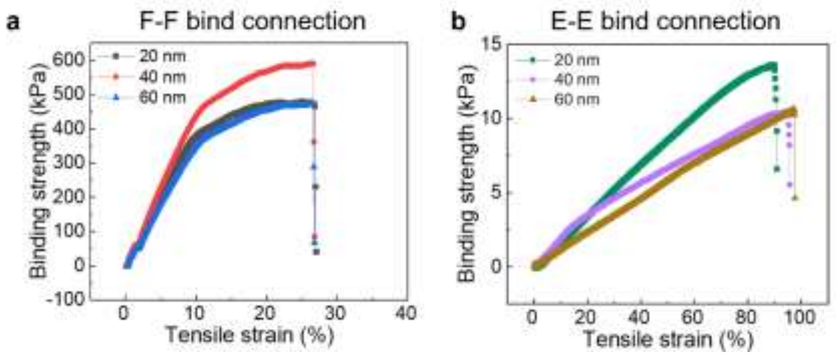

Supplementary Fig. 19: Binding strength of the Au electrodes on PPH-PET and PPH-PDMS substrates to form F-F binding and E-E binding, respectively. a, b, Binding strength of the Au electrodes by F-F and E-E connections with deposition thicknesses of 20 nm, 40 nm and 60 nm, respectively.

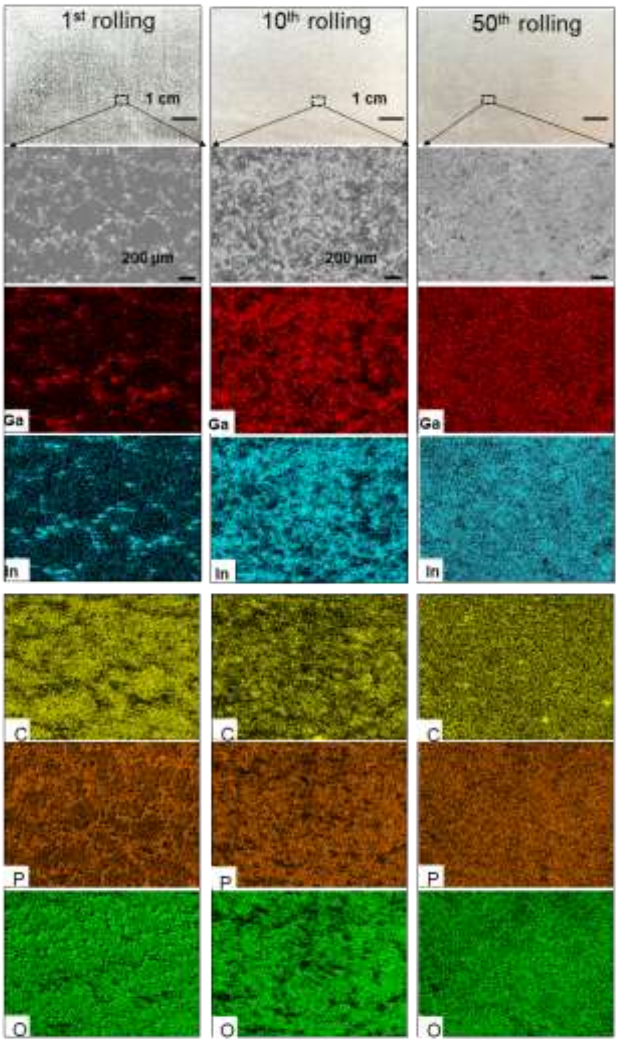

Supplementary Fig. 20: Elemental distribution images of Ga, In, C, P and O of surface passivated LM by a PPH layer under 1st, 10th and 50th rolling.

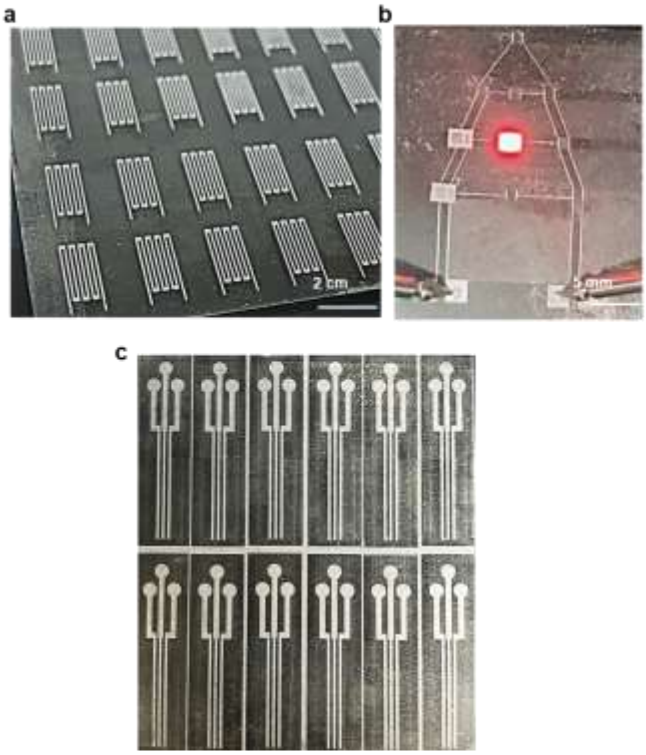

Supplementary Fig. 21: Photos of conductive LM electrodes obtained by roll-printing and selective laser patterning. A red μ-led was lighted by the LM electrodes.

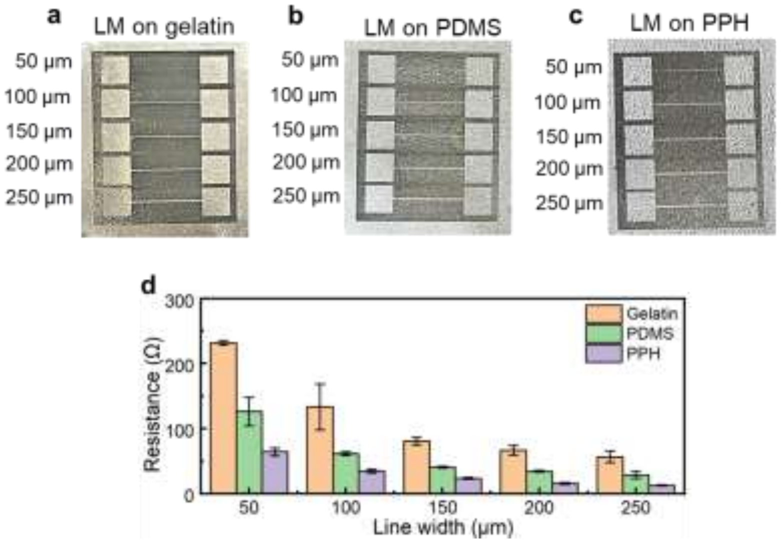

Supplementary Fig. 22: Photos of conductive LM electrodes with different line widths under laser patterning. a, b, c, The LM patterns with different line widths by roll-printing on gelatin, PDMS and PPH hydrogel. **d,** Line resistances of LM electrodes on gelatin, PDMS and PPH after laser patterning. Data were presented as means ±SD, n=3.

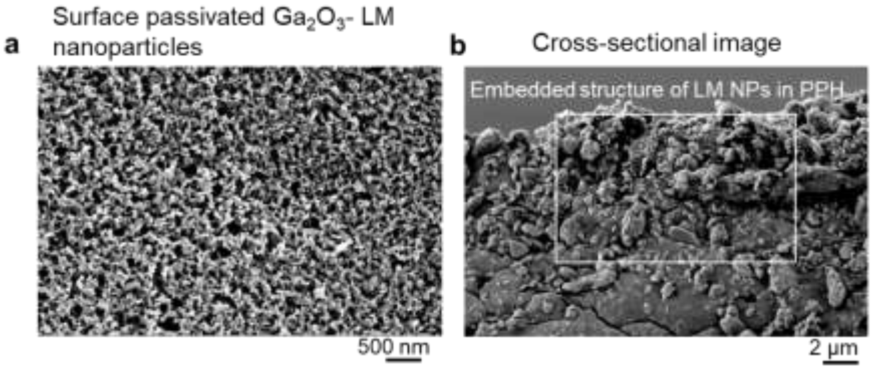

Supplementary Fig. 23: Images that displayed the embedded structures of Ga_2O_3-LM nanoparticles in PPH. a, b, Frontal and cross-sectional SEM images of surface passivated Ga_2O_3-LM nanoparticles after 50 times of rolling.

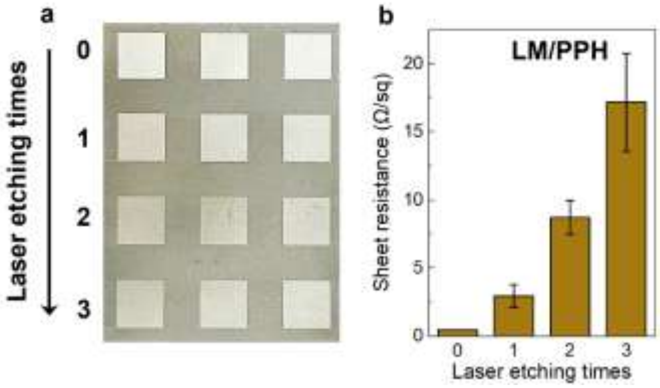

Supplementary Fig. 24: The remained LM electrodes on PPH-PDMS after 3 times of etching by a nanosecond UV laser system. a, Image of the LM electrodes maintained on PPH surface after laser etching at different times. **b,** Sheet resistance of squared LM electrodes on PPH surface after laser etching at different times. Data were presented as means ±SD, n=3.

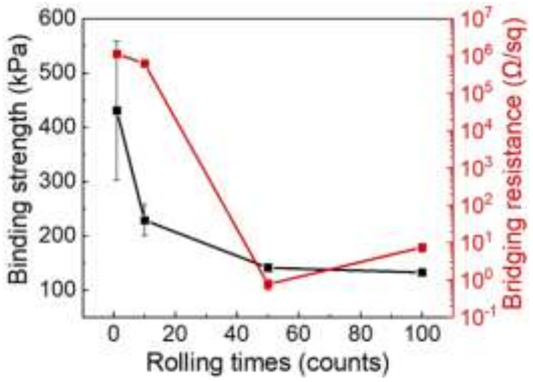

Supplementary Fig. 25: Binding strength and bridging resistance of PPH passivated LM surfaces at different roll-printing times. Data were presented as means ±SD, n=3.

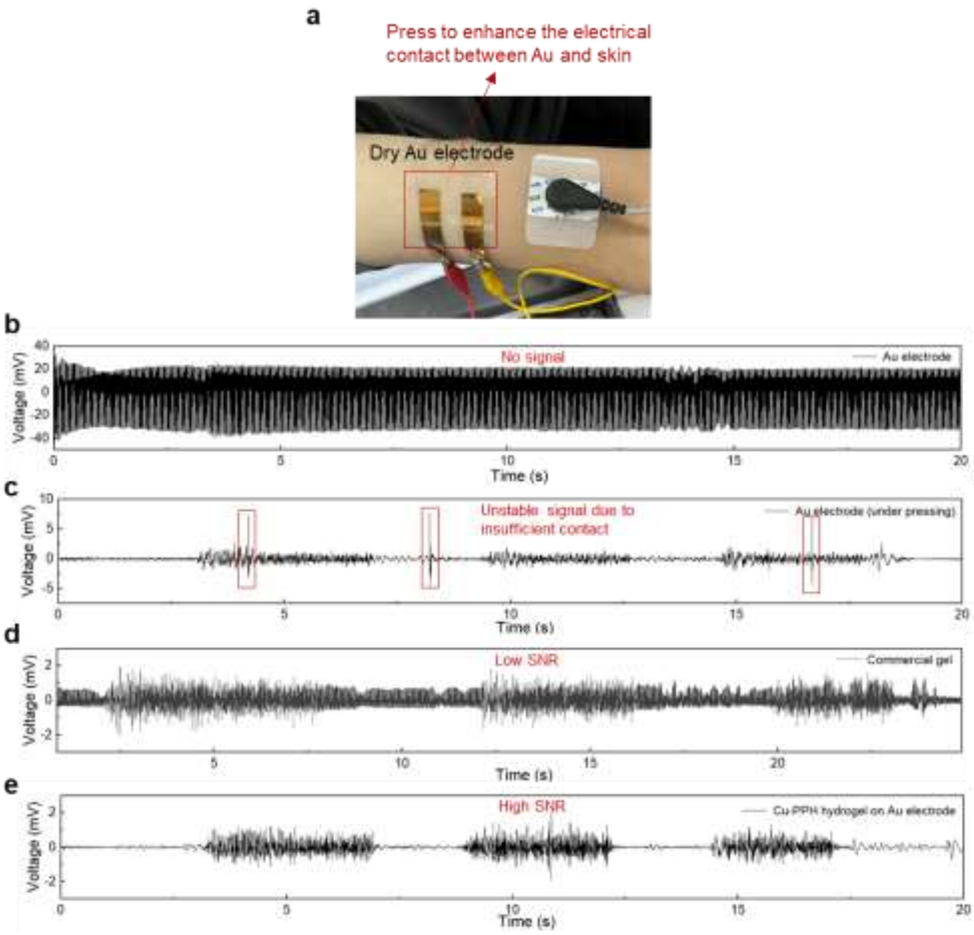

Supplementary Fig. 26: EMG signals recorded on bicipital muscle of right arm of an adult by using different dry and wet electrodes. a, Photo of dry Au electrodes on the arm. b, c, EMG signals recorded using Au electrodes under naturally attachment and pressing **d, e,** EMG signals recorded using commercial Ag/AgCl gel and the bidirectional interface of a Cu-PPH hydrogel and the Au electrode.

Captions for Supplementary Videos:
Supplementary Video 1: Adhesion of a Cu-PPH hydrogel on PDMS.

Supplementary Video 2: Adhesion of a Cu-PPH hydrogel on human skin.

Supplementary Video 3: Compression test of two pieces of Cu billets under strong surface chelation.

Supplementary Video 4: Strong binding strength of face-to-face attached Au-PPH-PET films.

Supplementary Video 5: A soft Cu-PPH hydrogel attached on the skin of a human hand. The soft Cu-PPH hydrogel was strongly adhered on to the skin even under stretching.

Supplementary Video 6: Laser patterning process of the surface chelated LM-PPH on a PET substrate after roll-printing. The unwanted area of LM-PPH was selectively cleaned by a nanosecond UV laser.
