# Supplementary material for: Metal–hydrogel chelation interfaces for ultrasoft and bidirectional bioelectronics
Source: Natl Sci Rev. 2025 Sep 18;12(11):nwaf399. doi: 10.1093/nsr/nwaf399 (PMC12618001; doi:10.1093/nsr/nwaf399)
Supplement: nwaf399_Supplemental_Files [file nwaf399_supplemental_files.zip › Supporting information.docx]

**Supporting information**

**Metal-Hydrogel Chelation Interfaces for**

**Ultrasoft and Bidirectional Bioelectronics**

Yuyao Lu^1*^, Ziguan Jin^1^, Yihui Jian^1^, Depeng Kong^1^, Hao Zhou^1^, Yuhong Xu^1^, Ruijue Cao^2^, Zhuoheng Xia^3^, Fan Yang^2^, Qianglong Wu^4^, Yang Gao^4^, Aoran Cui^5^, Shikuan Yang^5^, Nenggan Zheng^6^, Junhyuk Bang^7^, Geng Yang^1,8*^, Seung Hwan Ko^7^, Huayong Yang^1^, Kaichen Xu^1*^

^1^State Key Laboratory of Fluid Power and Mechatronic Systems, School of Mechanical Engineering, Zhejiang University, Hangzhou 310027, China

^2^Center for Plastic & Reconstructive Surgery, Department of Stomatology, Zhejiang Provincial People's Hospital, Affiliated People's Hospital, Hangzhou Medical College, Hangzhou, Zhejiang, China

^3^School of Stomatology, Zhejiang Chinese Medical University, Hangzhou, Zhejiang, China

^4^Center for X-mechanics, Department of Engineering Mechanics, Zhejiang University, Hangzhou, China

^5^Institute for Composites Science Innovation, School of Materials Science and Engineering, Zhejiang University, Hangzhou 310027, China

^6^State Key Laboratory of Brain-Machine Intelligence and the Qiushi Academy for Advanced Studies, Zhejiang University, Hangzhou 310027, China

^7^Department of Mechanical Engineering, Seoul National University, Seoul, Korea

^8^Zhejiang Key Laboratory of Intelligent Robot for Operation and Maintenance, Hangzhou 310000, China

^*^Email addresses of corresponding authors: lu_yy@zju.edu.cn; [yanggeng@zju.edu.cn](mailto:yanggeng@zju.edu.cn); [xukc@zju.edu.cn](mailto:xukc@zju.edu.cn)

**
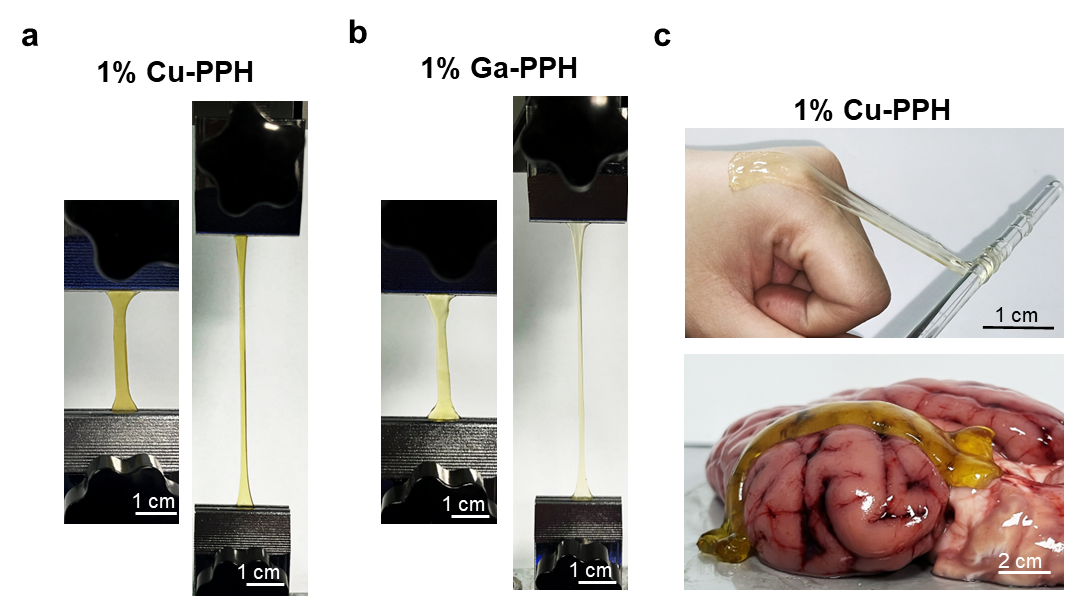
**

**Supplementary Fig. 1: Photo images of internally chelated soft hydrogels. a, b,** Photos of stretched Cu-PPH and Ga-PPH hydrogels. **c,** Photos of soft Cu-PPH hydrogel attached on skin and brain tissue.

**
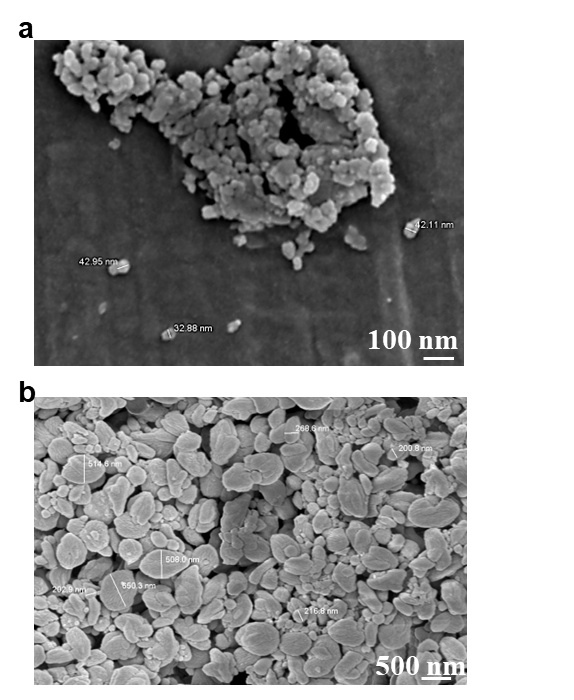
**

**Supplementary Fig. 2: Scanning electron microscope (SEM) images of the (a) Cu nanoparticles and (b) Ga_2_O_3_-LM microspheres.**


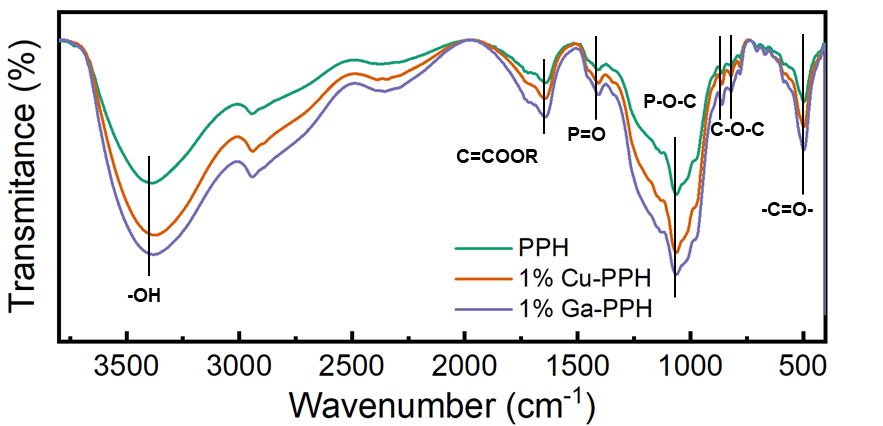


**Supplementary Fig. 3: Infrared (IR) spectra of** **PPH hydrogels, 1% Cu-PPH hydrogels and 1% Ga-PPH hydrogels.**


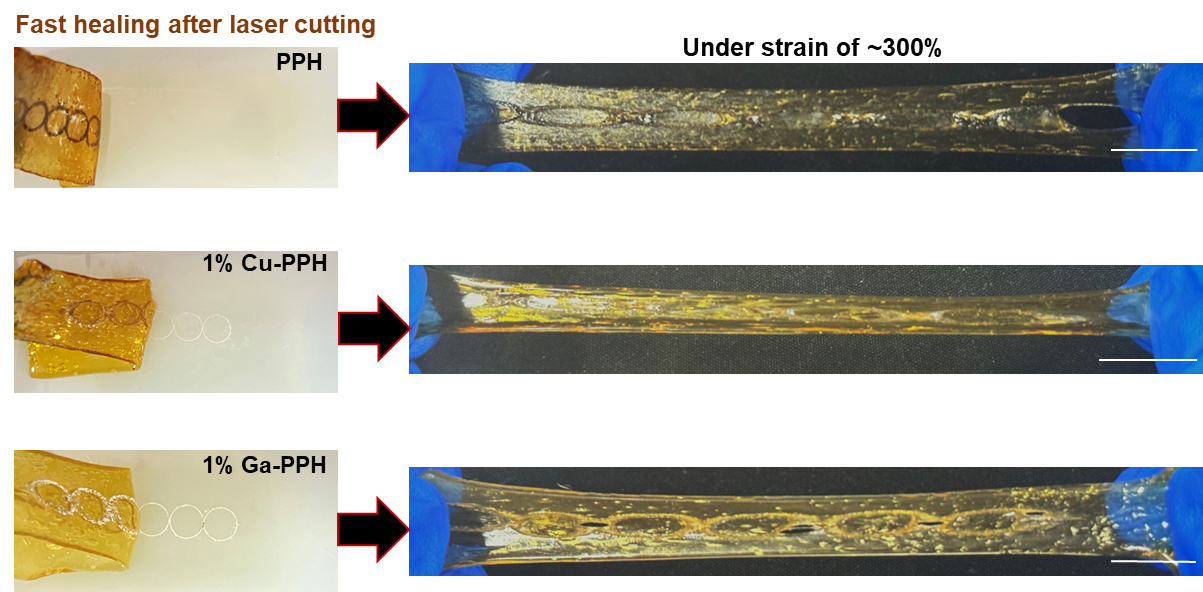


**Supplementary Fig. 4: Self-healing property of PPH, 1% Cu-PPH and 1% Ga-PPH hydrogels under laser cutting.** The images presented the stretchability of these hydrogels with fast-healing performances under laser cutting. Scale bar: 1 cm.


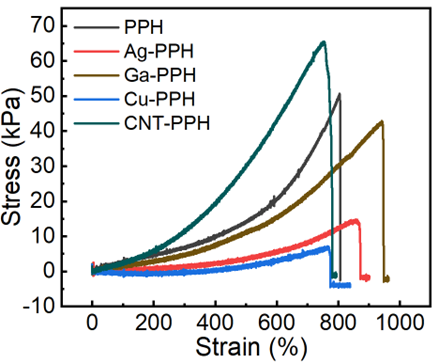


**Supplementary Fig. 5: Tensile performances of different metal-chelated hydrogels and the CNTs-doped hydrogel.** Metallic nanomaterials include Ag nanowires, Cu nanoparticles and Ga_2_O_3_-LM microspheres.


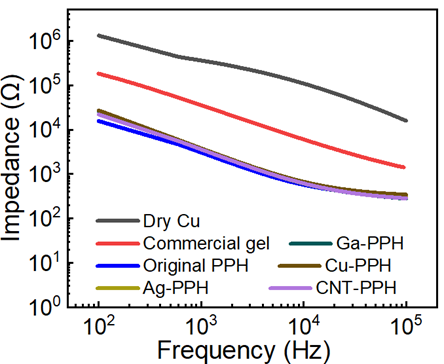


**Supplementary Fig. 6: On-skin contact impedances of dry Cu, commercial gel, PPH hydrogel, CNTs-doped PPH hydrogel as well as metal-chelated PPH hydrogels.** Metallic nanomaterials include Ag nanowires, Cu nanoparticles and Ga_2_O_3_-LM microspheres.


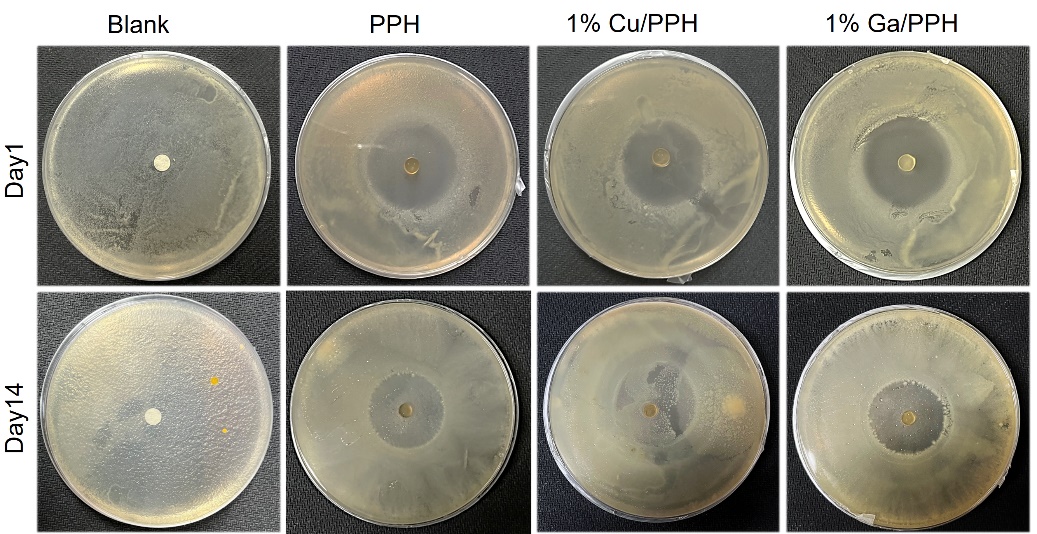


**Supplementary Fig. 7: Anti-bacterial performances of blank filter paper, PPH, 1% Cu-PPH and 1% Ga-PPH that were cut into circle pieces with a diameter of 6 mm.** The inhibition zone pictures in the first and second rows were recorded on day 1 and day 14, respectively.


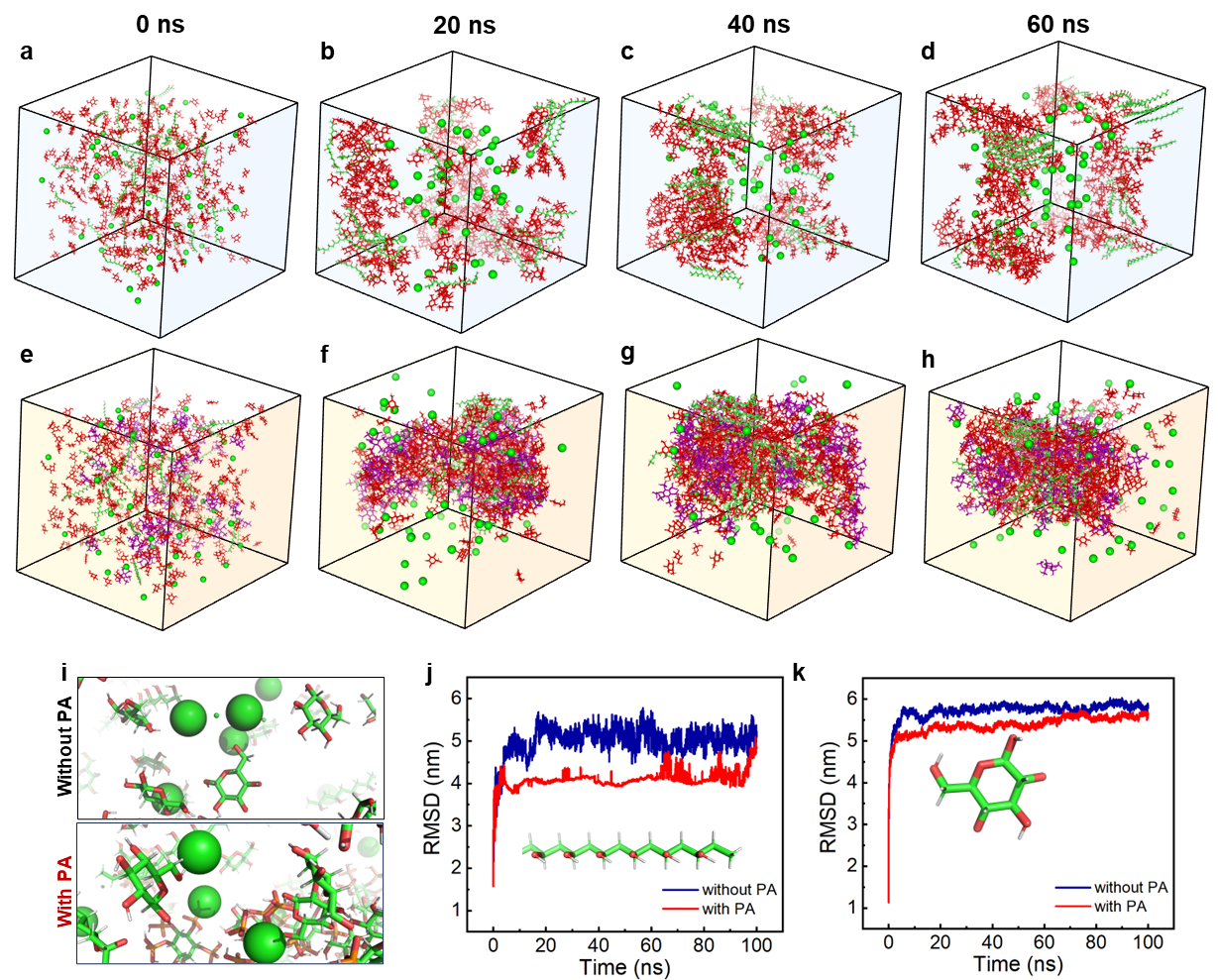


**Supplementary Fig. 8:** **Molecular dynamics (MD) simulation results of Cu-PPH hydrogels wo/w phytic acid (PA).** Snapshots of Cu-PPH composite hydrogel without chelation agent of PA at 0 ns (**a**), 20 ns (**b**), 40 ns (**c**), 60 ns (**d**), respectively. Snapshots of Cu chelated PPH hydrogel with PA at 0 ns (**e**), 20 ns (**f**), 40 ns (**g**), 60 ns (**h**), respectively. **i,** Magnified snapshots of different components distributed around Cu^2+^ in the presence and absence of PA. **j,** Radial distribution function (RDF) g(r) of Cu^2+^ and different molecules for the PPH hydrogels in the presence and absence of PA. **k,** Root mean square deviation (RMSD) results of PVA in the presence and absence of PA within 100 ns.


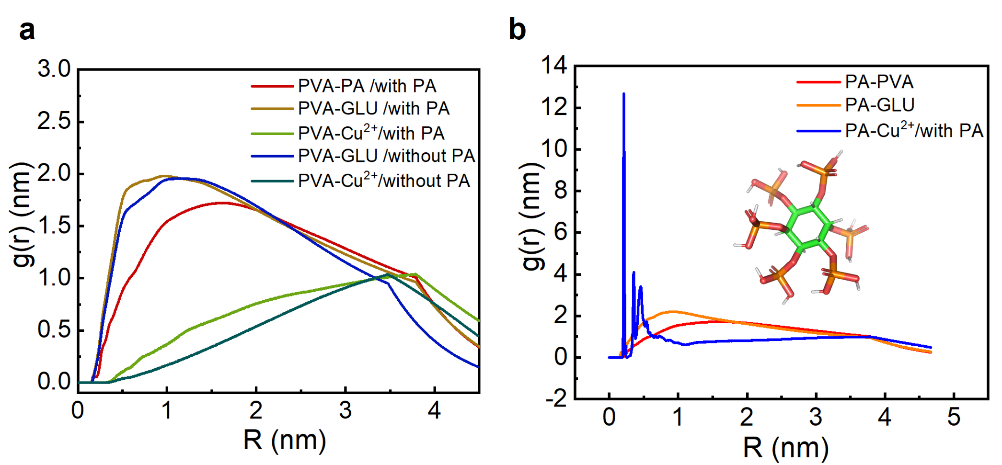


**Supplementary Fig. 9: Radial distribution function (RDF) results of PPH hydrogels in the presence and absence of PA. a,** g(r) of PVA and different molecules**. b,** g(r) of PA and different molecules.


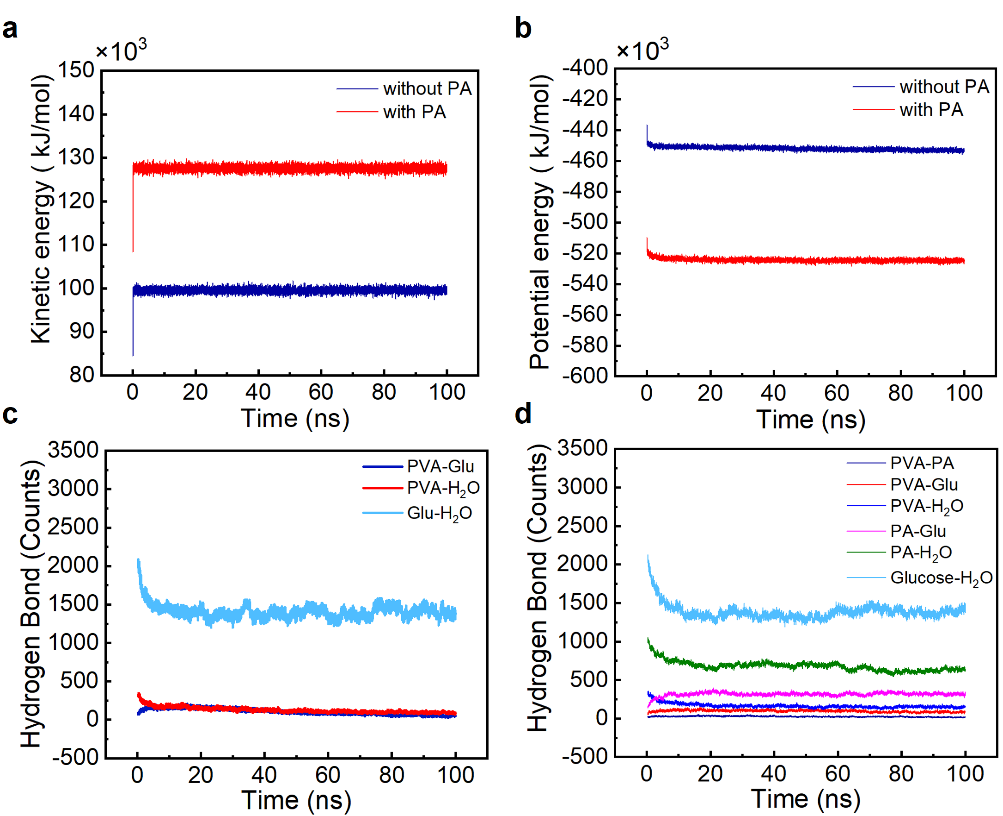


**Supplementary Fig. 10: Molecular dynamics (MD) calculation results of the two hydrogel systems without and with PA. a, b,** Kinetic energy and potential energy of the two hydrogel systems simulated in 100 ns. **c, d**, Number of hydrogen bonds formed in the two hydrogel systems.


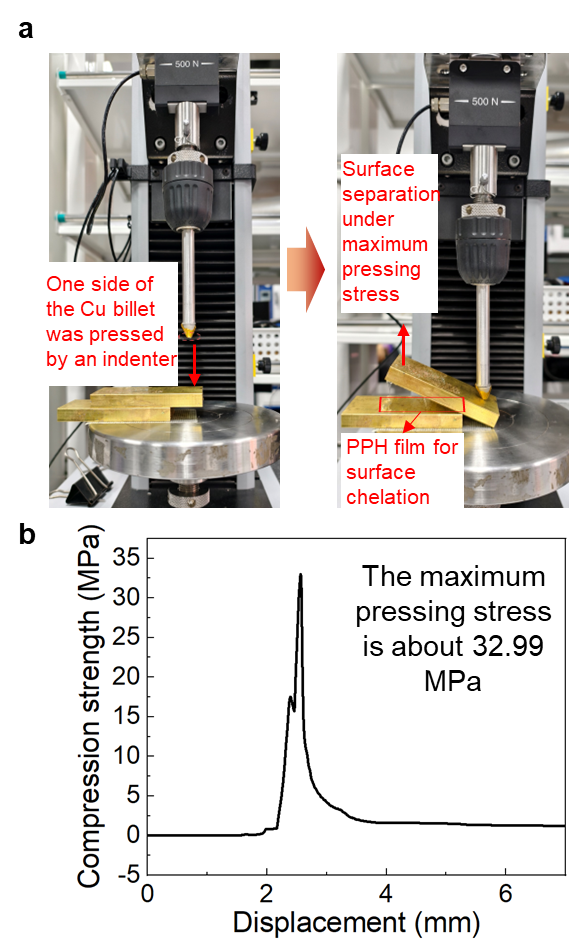


**Supplementary Fig. 11: Adhesion strength of two Cu billets under surface chelation a,** Photos of the compression test. **b,** The maximum pressing stress measured for separating two Cu billets under surface chelation.


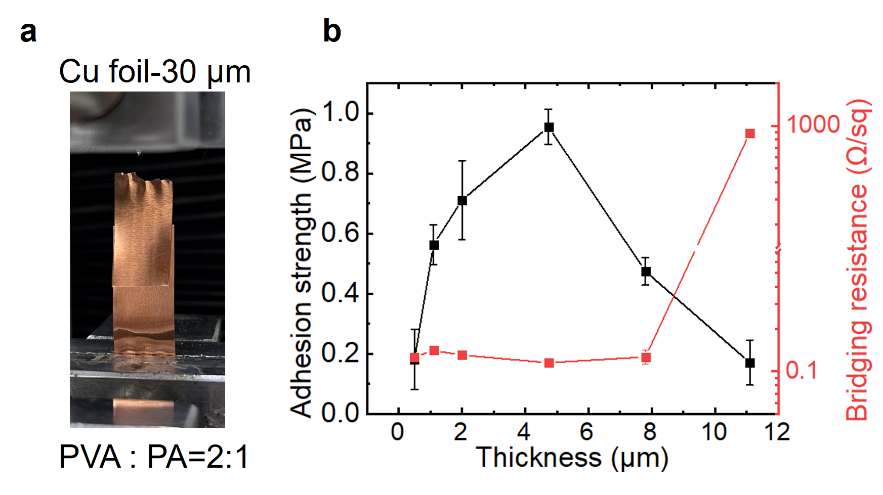


**Supplementary Fig. 12: Adhesion strength of two pieces of Cu foils (30 μm) with different thicknesses of PPH adhesive layer. a,** Photo of a fractured Cu foil strongly connected to another Cu foil via surface chelation by PPH hydrogel. **b,** Adhesion strength and bridging resistance between the two Cu foils by surface chelation. Data were presented as means ±SD, n=3.


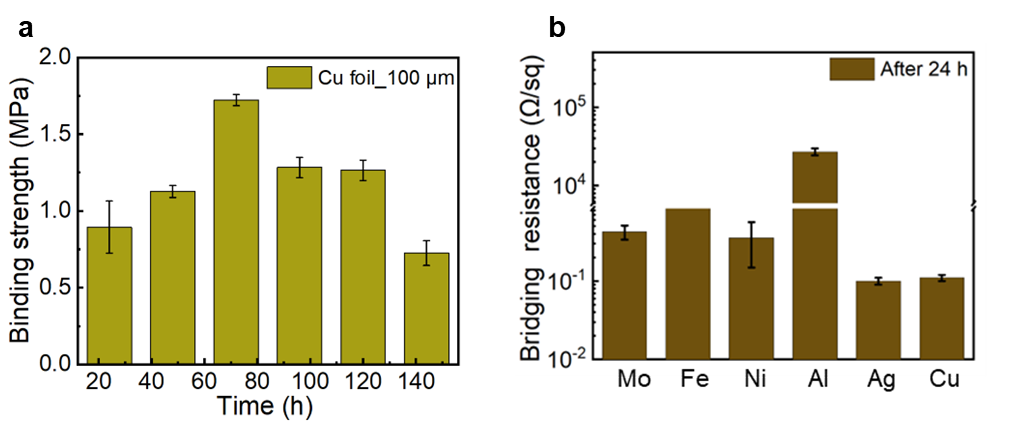


**Supplementary Fig. 13: Long-time binding strength and bridging resistance of different metal foils using the PPH as the surface chelation agent and adhesion layer. a,** Binding strength of two pieces of Cu foils investigated by lap shear tests under different chelation times. **b,** The bridging resistance of different metal foils after 24 h of surface chelation. Data were presented as means ±SD, n=3.


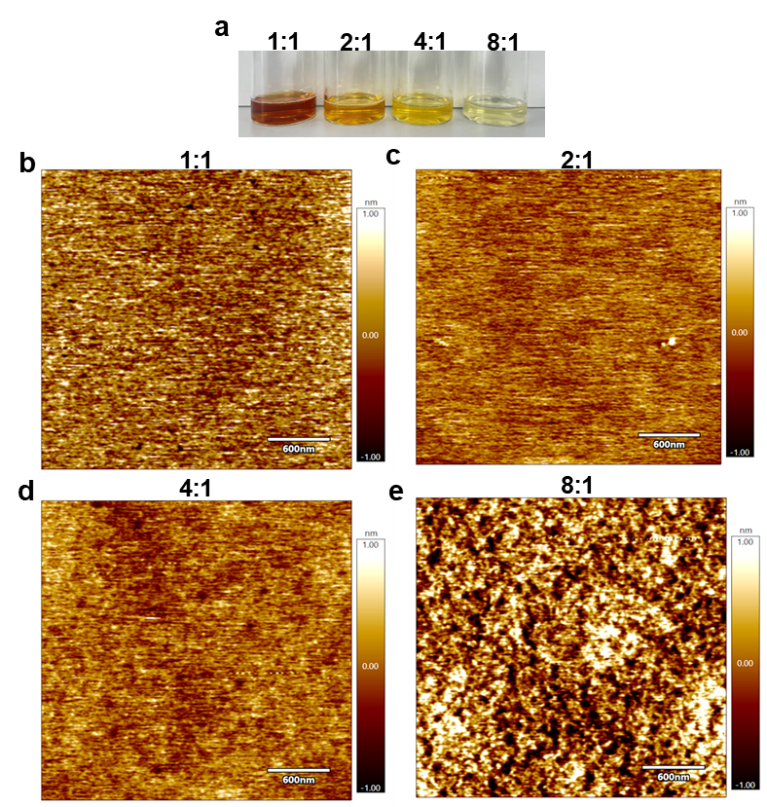


**Supplementary Fig. 14: Atomic force microscopy (AFM) images of PPH hydrogels with different ratios of PVA and PA. a,** Photo of partially crosslinked PPH solutions with different ratios of PVA and PA. **b, c, d, e,** Phase maps of PPH hydrogels with different ratios of PVA and PA.


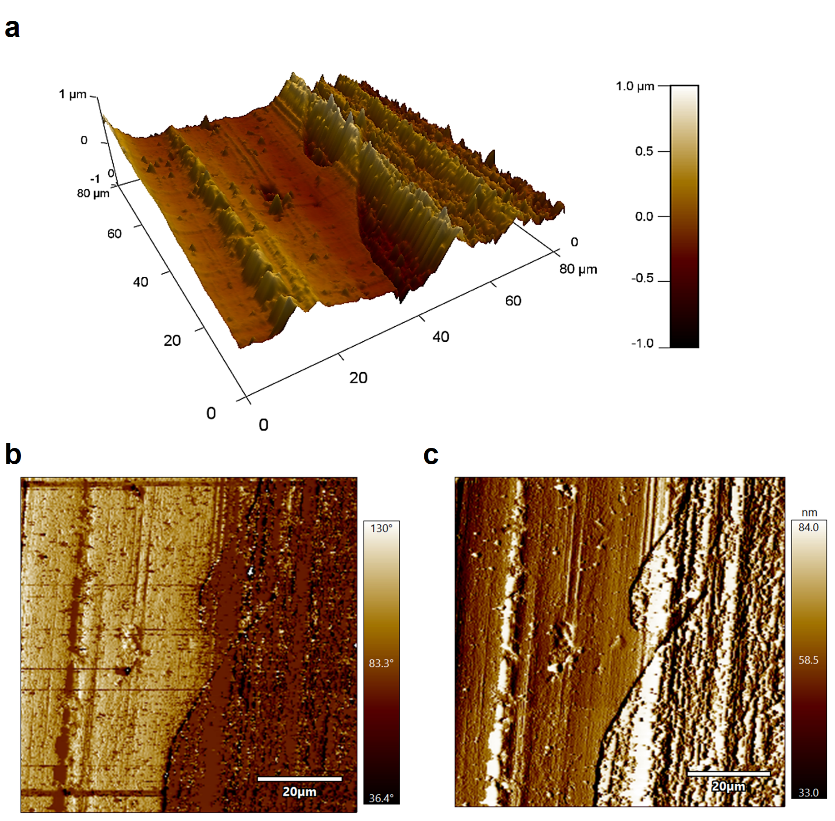


**Supplementary Fig. 15: Surface topography and roughness maps of Cu foil before and after surface treatments by a thin layer of PPH. a,** 3D mapping result of the pristine Cu (left area) and PPH-treated Cu (right area). **b, c,** Phase and amplitude maps of Cu foils before and after treatment by a thin layer of PPH.


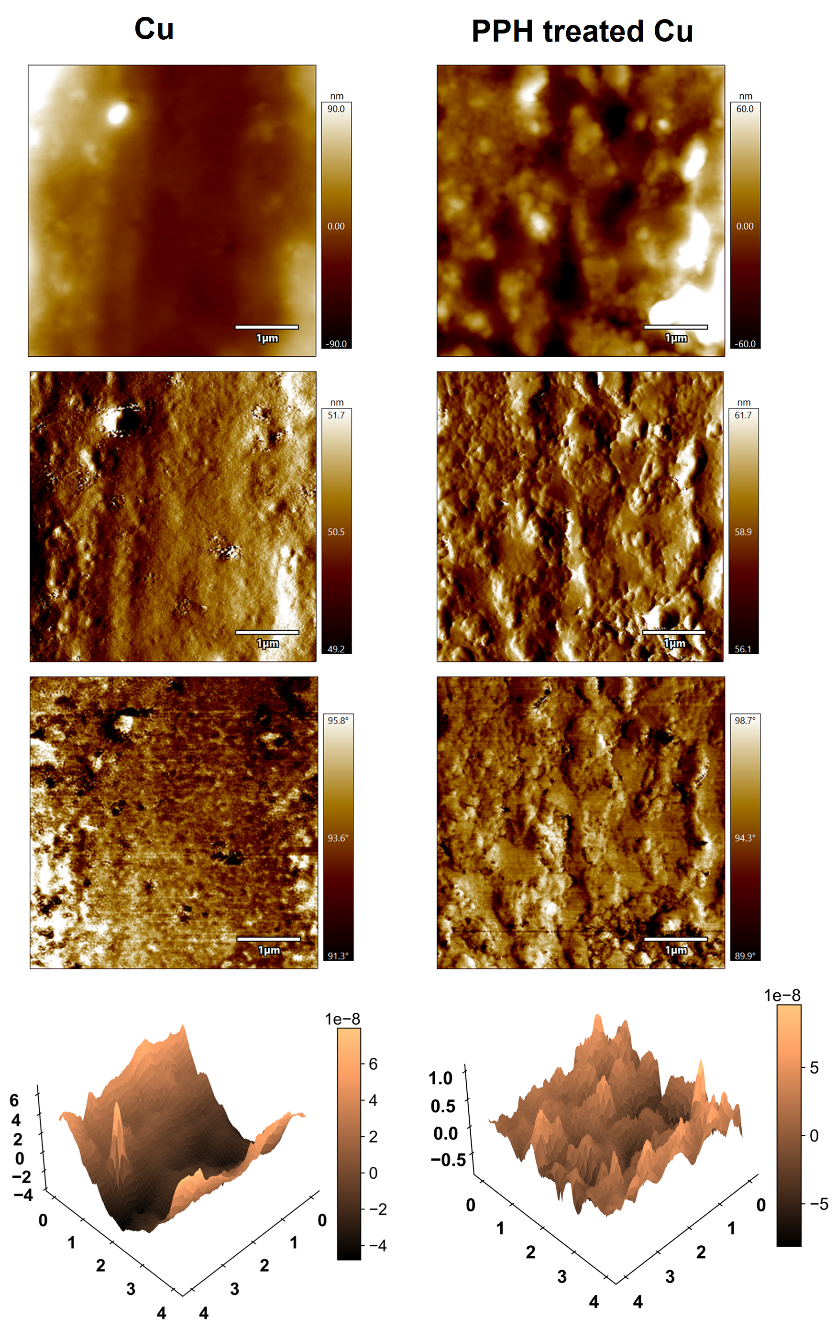


**Supplementary Fig. 16: Surface topography, phase and amplitude maps of Cu foils before and after treatments by a thin layer of PPH.**


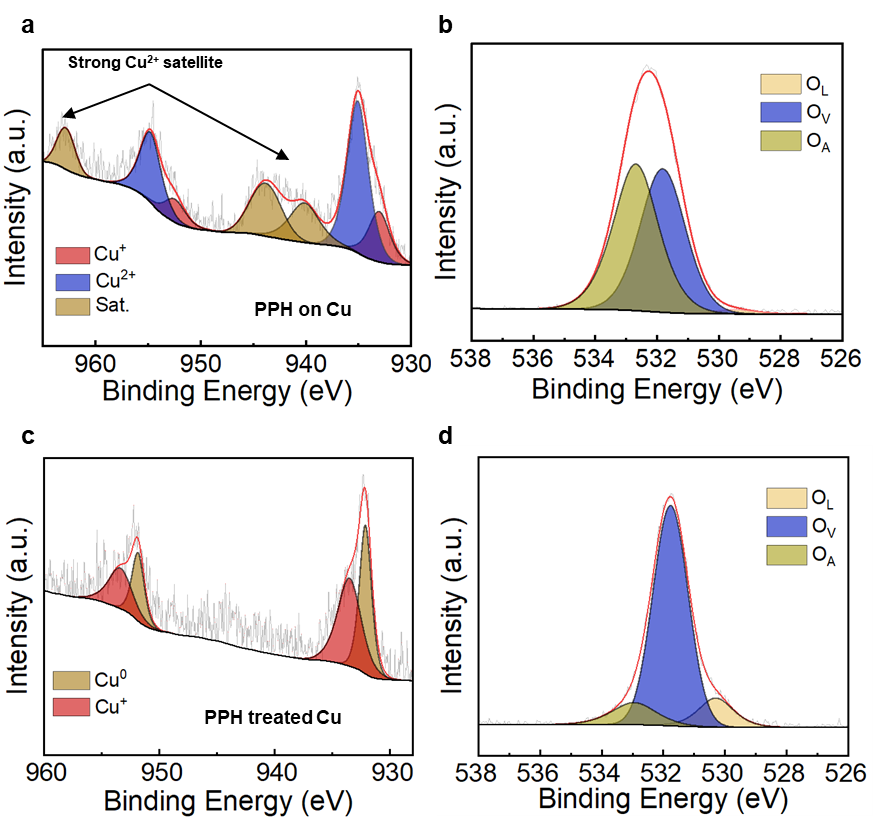


**Supplementary Fig. 17: The x-ray photoelectron spectroscopy (XPS) results of Cu and O on surfaces of PPH coated Cu and PPH treated Cu after removing PPH. a, b,** XPS spectra of Cu and O on PPH coated Cu. **c, d,** XPS spectra of Cu and O on PPH treated Cu.


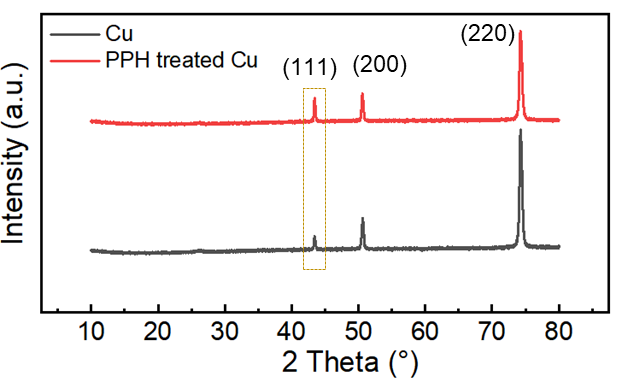


**Supplementary Fig. 18: X-ray Diffraction (XRD) results of pristine Cu and PPH treated Cu.**


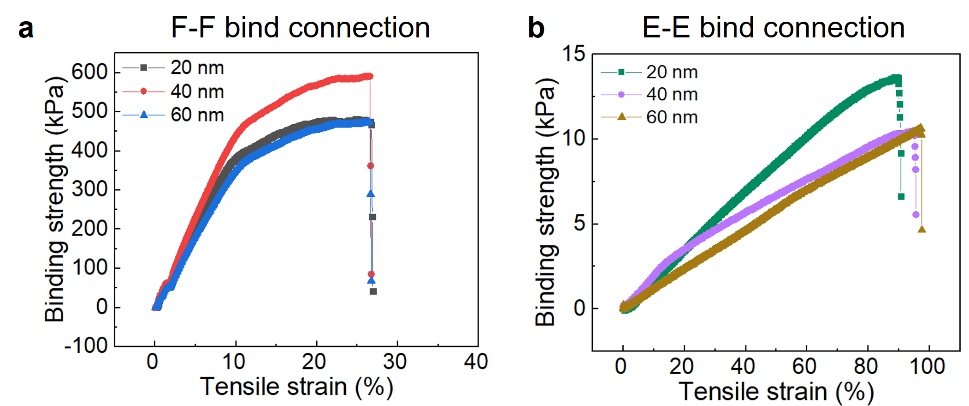


**Supplementary Fig. 19: Binding strength of the Au electrodes on PPH-PET and PPH-PDMS substrates to form F-F binding and E-E binding, respectively. a, b,** Binding strength of the Au electrodes by F-F and E-E connections with deposition thicknesses of 20 nm, 40 nm and 60 nm, respectively.


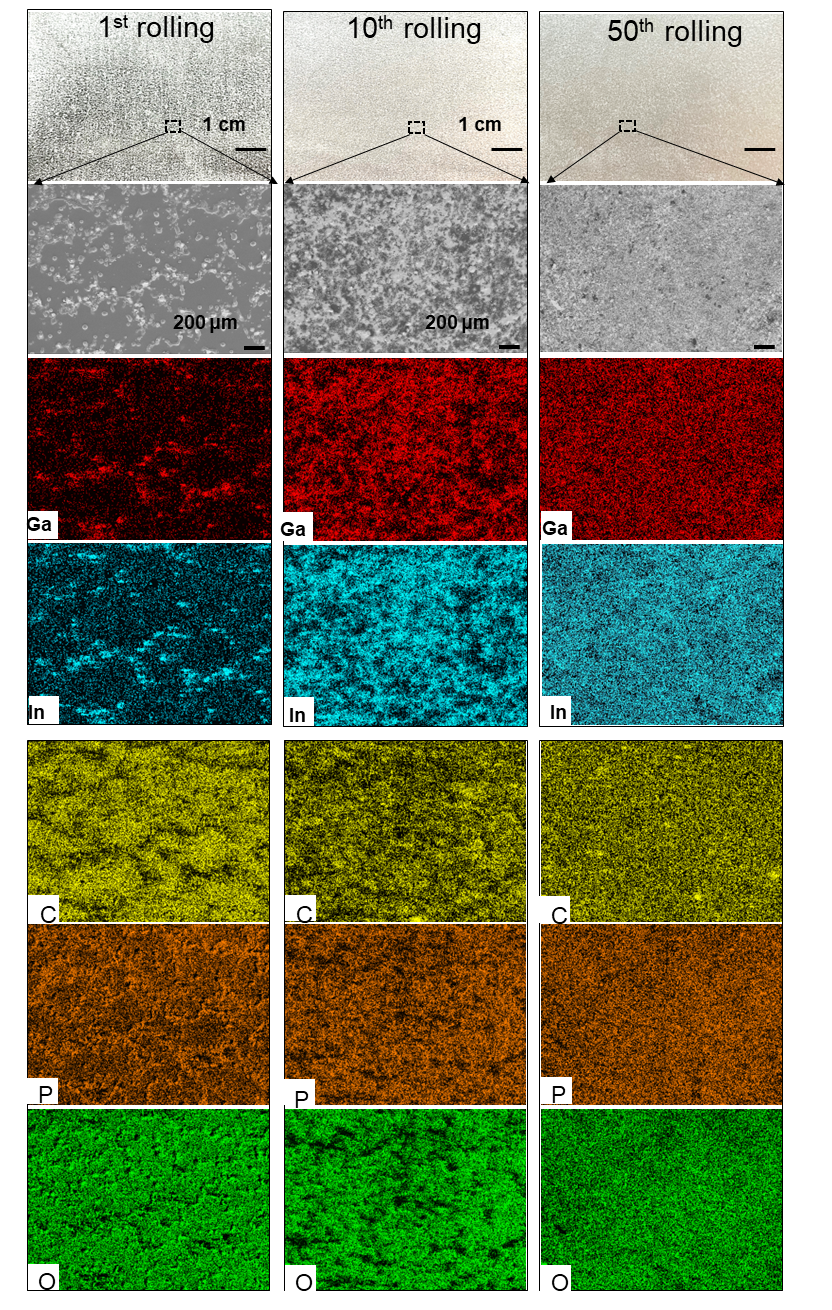


**Supplementary Fig. 20: Elemental distribution images of Ga, In, C, P and O of surface passivated LM by a PPH layer under 1^st^, 10^th^ and 50^th^ rolling.**


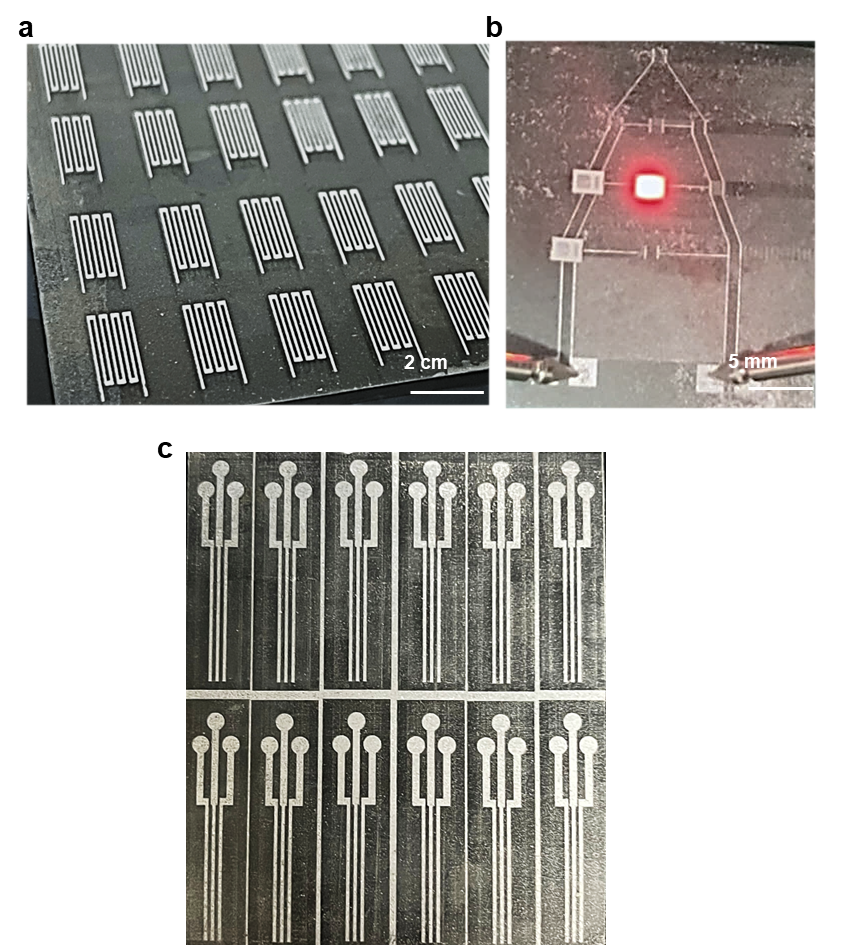


**Supplementary Fig. 21: Photos of conductive LM electrodes obtained by roll-printing and selective laser patterning.** A red μ-led was lighted by the LM electrodes.


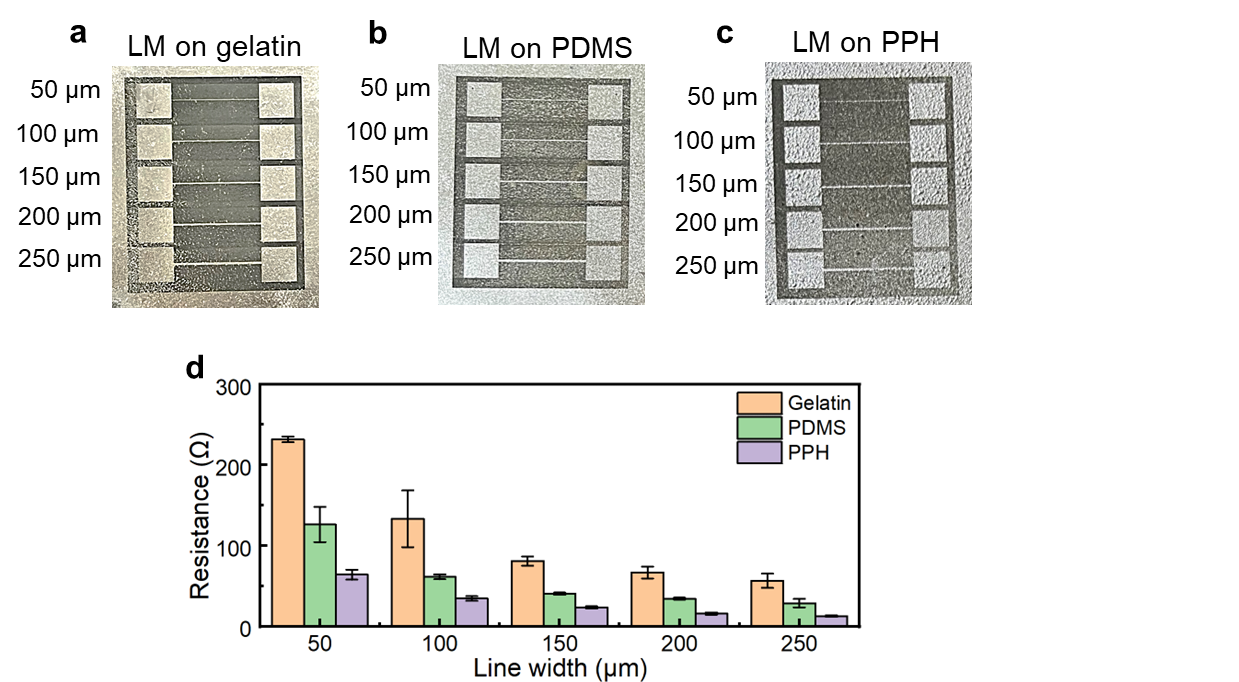


**Supplementary Fig. 22: Photos of conductive LM electrodes with different line widths under laser patterning. a, b, c,** The LM patterns with different line widths by roll-printing on gelatin, PDMS and PPH hydrogel. **d,** Line resistances of LM electrodes on gelatin, PDMS and PPH after laser patterning. Data were presented as means ±SD, n=3.


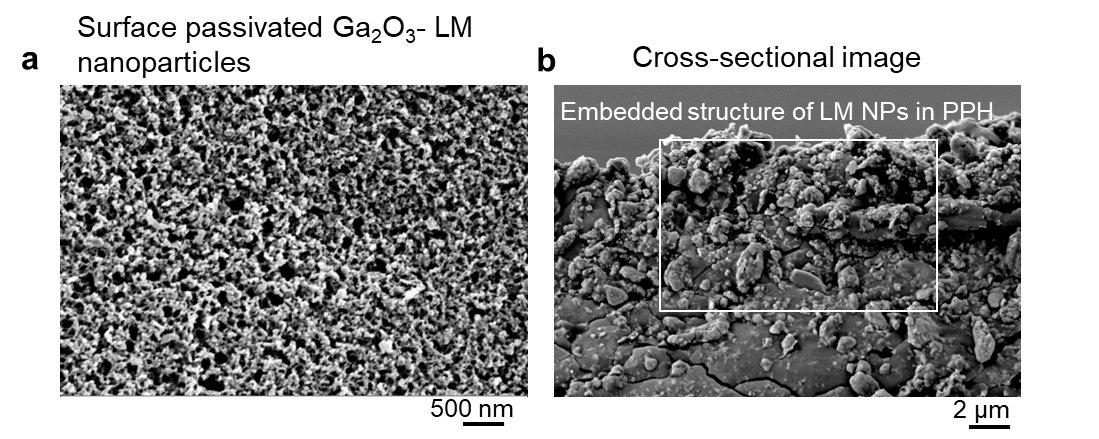


**Supplementary Fig. 23: Images that displayed the embedded structures of Ga_2_O_3_-LM nanoparticles in PPH. a, b,** Frontal and cross-sectional SEM images of surface passivated Ga_2_O_3_-LM nanoparticles after 50 times of rolling.


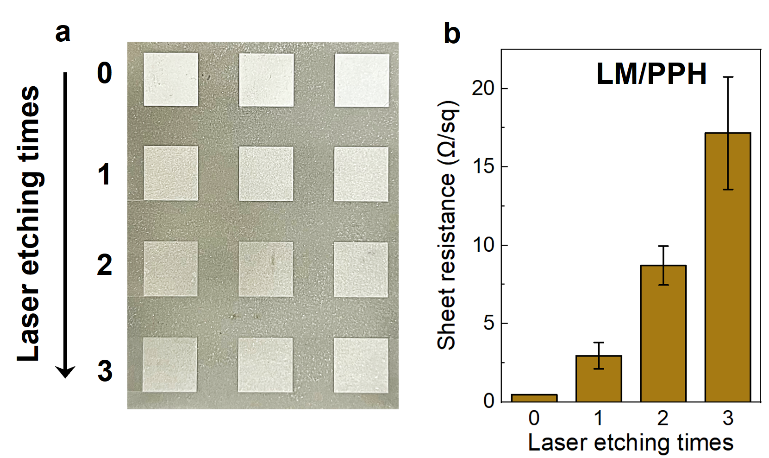


**Supplementary Fig. 24: The remained LM electrodes on PPH-PDMS after 3 times of etching by a nanosecond UV laser system. a,** Image of the LM electrodes maintained on PPH surface after laser etching at different times. **b,** Sheet resistance of squared LM electrodes on PPH surface after laser etching at different times. Data were presented as means ±SD, n=3.


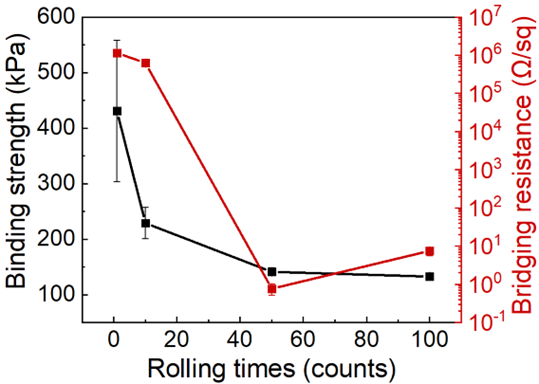


**Supplementary Fig. 25: Binding strength and bridging resistance of PPH passivated LM surfaces at different roll-printing times.** Data were presented as means ±SD, n=3.


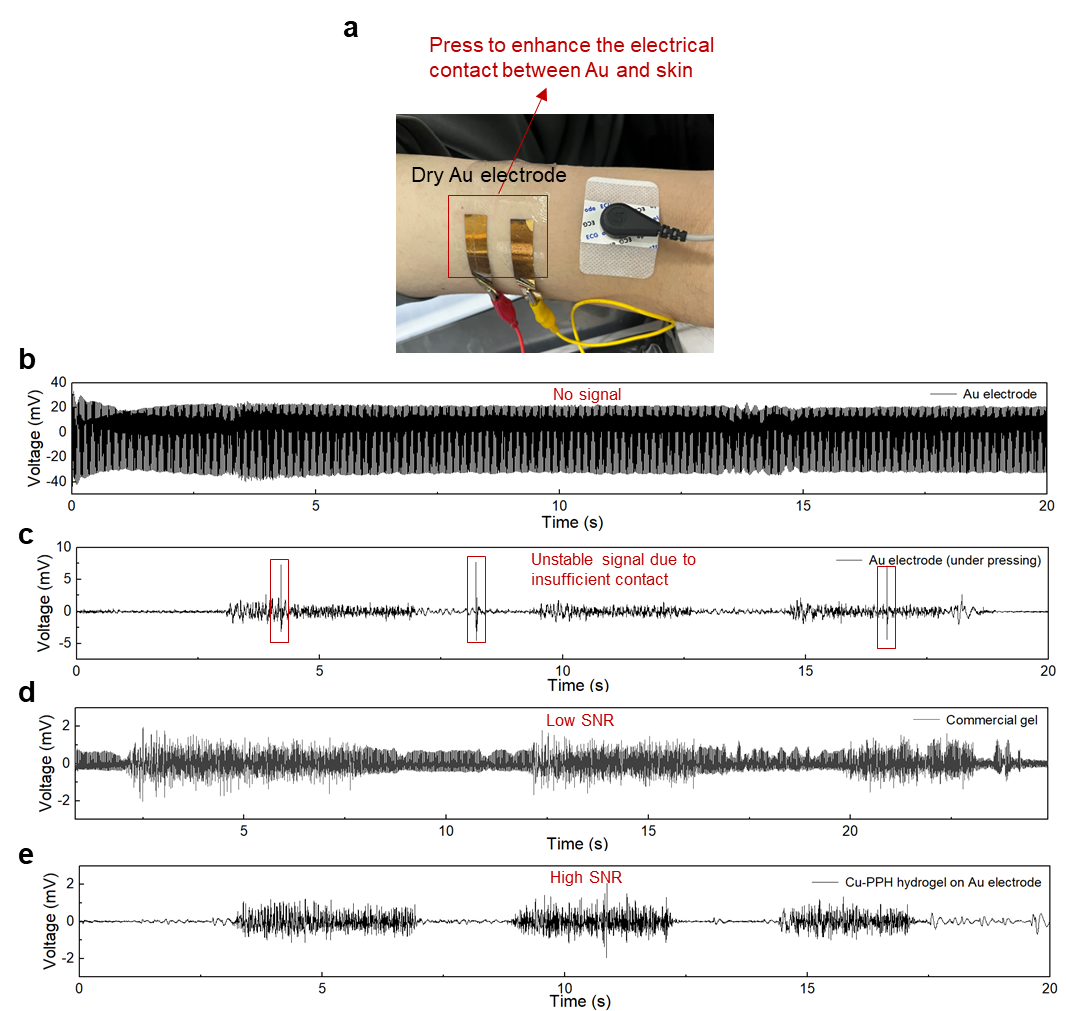


**Supplementary Fig. 26: EMG signals recorded on bicipital muscle of right arm of an adult by using different dry and wet electrodes. a, Photo of dry Au electrodes on the arm. b, c,** EMG signals recorded using Au electrodes under naturally attachment and pressing **d, e,** EMG signals recorded using commercial Ag/AgCl gel and the bidirectional interface of a Cu-PPH hydrogel and the Au electrode.

**Captions for Supplementary Videos:**

**Supplementary Video 1: Adhesion of a Cu-PPH hydrogel on PDMS.**

**Supplementary Video 2: Adhesion of a Cu-PPH hydrogel on human skin.**

**Supplementary Video 3: Compression test of two pieces of Cu billets under strong surface chelation.**

**Supplementary Video 4: Strong binding strength of face-to-face attached Au-PPH-PET films.**

**Supplementary Video 5:** **A soft Cu-PPH hydrogel attached on the skin of a human hand.** The soft Cu-PPH hydrogel was strongly adhered on to the skin even under stretching.

**Supplementary Video 6:** **Laser patterning process of the surface chelated LM-PPH on a PET substrate after roll-printing.** The unwanted area of LM-PPH was selectively cleaned by a nanosecond UV laser.
